# Pet Wellness and Vitamin A: A Narrative Overview

**DOI:** 10.3390/ani14071000

**Published:** 2024-03-25

**Authors:** Yauheni Shastak, Wolf Pelletier

**Affiliations:** Nutrition & Health Division, BASF SE, 67063 Ludwigshafen am Rhein, Germany

**Keywords:** pets, dog, cat, vitamin A, health, well-being

## Abstract

**Simple Summary:**

Vitamin A, a vital fat-soluble micronutrient, is indispensable for the health and well-being of companion animals, notably dogs and cats. Its multifaceted roles encompass crucial functions in vision, immune modulation, and reproductive health. Vitamin A is intricately involved in cellular differentiation, gene expression, and antioxidant defense mechanisms, exerting a profound influence on the overall physiological function. A deficiency in this essential vitamin can lead to a spectrum of health issues, including compromised vision, an impaired immune function, and reproductive abnormalities. A comprehensive understanding of the mechanisms involved in the absorption, cellular uptake, and metabolic pathways of vitamin A is crucial for optimizing the nutrition of companion animals. Further research on retinoids is essential to deepen our understanding and to refine dietary recommendations tailored to the unique needs of companion animals, thereby ensuring their optimal health and vitality.

**Abstract:**

The health of companion animals, particularly dogs and cats, is significantly influenced by nutrition, with vitamins playing a crucial role. Vitamin A, in particular, is indispensable, with diverse roles ranging from vision to immune modulation and reproduction. Despite its importance, the metabolism and dietary requirements of vitamin A in companion animals remain complex and not fully understood. This review provides a comprehensive overview of the historical perspective, the digestion, the metabolism, the physiological roles, the deficiency, the excess, and the interactions with other micronutrients of vitamin A in companion animals. Additionally, it highlights future research directions and gaps in our understanding. Insights into the metabolism of vitamin A in companion animals, personalized nutrition strategies based on genetic variability, longitudinal studies tracking the status of vitamin A, and investigations into its immunomodulatory effects are crucial for optimizing pet health and wellness. Furthermore, understanding the stability and bioavailability of vitamin A in pet food formulations is essential for ensuring the provision of adequate micronutrients. Overall, this review underscores the importance of vitamin A in companion animal nutrition and the need for further research to enhance our understanding and to optimize dietary recommendations for pet health and well-being.

## 1. Introduction

In the important domain of companion animal health, particularly concerning species such as dogs and cats, there is an increasing emphasis on the vital role of nutrition, particularly in relation to vitamins. Veterinarians, pet owners, and researchers are progressively acknowledging the significance of these organic compounds in animal well-being and longevity, given their indispensable role in physiological functions [1,2,3,4]. Vitamins, functioning as crucial components in the complex machinery of life, serve diverse roles, with vitamin A being notably versatile, involved in vision, immune system modulation, cellular differentiation, and reproduction [5]. Noteworthily, vitamin A is considered by many veterinary nutritionists to be the most important vitamin [6]. The term “vitamin A” encompasses three chemical compounds: retinol, retinal, and retinoic acid (all-trans retinoic acid, ATRA) [7].

The profound bond between humans and their pets transcends mere ownership, evolving into a shared journey of companionship and caregiving. As caretakers of their well-being, it is crucial for owners to comprehend the fundamental nutritional elements contributing to the health of our pets [8]. The investigation of vitamin A in this context is not solely an academic endeavor; it delves into the foundational aspects of pet nutrition, exerting a substantial influence on their overall well-being [9,10].

Dietary sources serve as pathways through which pets obtain nutrients, and understanding the availability of vitamin A in various diets is crucial for ensuring optimal pet health [10,11]. The decisions about pet diets are not just about providing food; they are intentional choices that significantly impact the pets’ wellness by shaping their nutritional needs [12,13]. Vitamin A, sourced from various foods, becomes a critical consideration, whether obtained from animal products or as a precursor from plant-based sources. The presence and accessibility of vitamin A in pet diets establish the nutritional groundwork influencing a pet’s health trajectory [11,14].

It is crucial to recognize that dogs and cats, as obligate carnivores, have varying and unpredictable abilities to convert certain plant carotenoids like β-carotene into vitamin A, unlike many other animals such as poultry, livestock, and most wild animals [15]. This introduces a critical aspect into the dietary landscape of these carnivorous companions, where dependence on animal-derived or synthetic sources of vitamin A becomes pivotal for meeting their nutritional requirements [16]. This subtlety adds complexity to their nutritional needs, prompting pet owners and pet food producers to make informed choices tailored to the unique needs of pets. At its core, this review aims to answer the fundamental question: how does vitamin A contribute to the wellness of our pets? To unravel this inquiry, we embark on a scientific exploration that focuses on the following key facets:

Historical perspective on vitamin A in pet nutrition;
Digestion and metabolism of vitamin A;
Physiological role of vitamin A;
Vitamin A deficiency and excess in pets;
Interactions with other micronutrients;
Comparing vitamin A requirements: livestock vs. pets;
Future directions and research gaps.

By addressing these objectives, this review aims to deepen our understanding of the role of vitamin A in optimizing pet health and well-being. This knowledge can inform effective management strategies to ensure appropriate micronutrient intake and optimize dietary formulations for pet wellness.

## 2. Historical Perspective on Vitamin A in Pet Nutrition

The historical journey of vitamin A in pet nutrition spans over a century, marked by key discoveries and evolving dietary guidelines. From its identification as vital for growth to the current research on therapeutic uses, this history offers deep insights into scientific inquiry and nutritional advancements [17,18,19,20].

**Figure 1:**
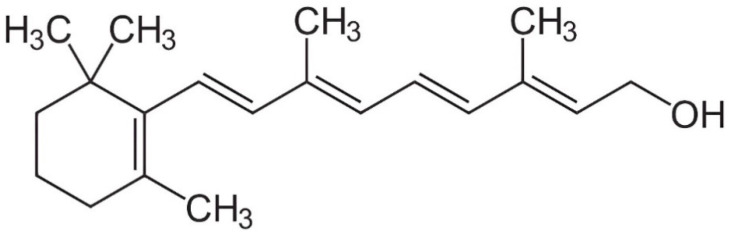
Structural formula for all-trans-retinol.

The discovery of vitamin A can be traced back to the early 20th century when researchers embarked on a quest to elucidate the causes of various nutritional deficiencies. In 1913, Elmer Vernon McCollum and Marguerite Davis conducted groundbreaking experiments on rats, revealing the existence of an essential factor vital for normal growth and health in animals [17]. This factor, later identified as vitamin A, emerged as a key player in preventing conditions such as night blindness and promoting overall well-being [21,22]. A significant stride in the practical application of vitamin A knowledge was its analytical determination in various foods and feeds [23]. The analytical determination of retinol began in the early 20th century, coinciding with the identification of vitamin A’s chemical structure by Paul Karrer in 1932 ([24]; Figure 1). Pivotal studies during this period focused on the isolation and purification of vitamin A from diverse natural sources, laying the foundation for subsequent analytical pursuits [25,26]. In 1937, Harry Holmes and Ruth Corbet isolated and crystallized vitamin A. The synthesis methods for vitamin A were later established through the research conducted by David Adriaan van Dorp and Jozef Ferdinand Arens in 1946, as well as by Otto Isler and his colleagues in 1947 [24].

These early discoveries sparked interest in understanding the dietary sources of vitamin A and its specific functions in different species, including companion animals [20]. Researchers delved into exploring the relationship between vitamin A and vision, reproduction, the immune function, and the integrity of epithelial tissues in dogs and cats [21,27].

As our understanding of vitamin A deepened, efforts were made to establish recommended dietary allowances to guide pet food formulation. The first nutritional requirements for dogs and cats were established in the 1960s and 1970s [28,29,30,31]. The National Academies of Sciences, Engineering, and Medicine (NASEM; formerly known as the National Research Council) later published updated recommendations for companion animals in 1985–1986, subsequently updated in 2006 [11,32,33]. These recommendations played a pivotal role in preventing vitamin deficiencies and associated health issues in companion animals. Mass production allowed for more consistent nutrient profiles, including vitamins, in commercial pet foods [34,35]. This era marked a shift from homemade diets to convenient, commercially prepared pet foods aimed at meeting essential nutritional requirements, including those of vitamin A.

The evolution of pet diets over the decades has influenced the formulation of vitamin A in commercial pet foods. With the transition towards commercially prepared pet foods, manufacturers have incorporated vitamin A from various sources to meet the specific needs of dogs and cats [16]. The inclusion of synthetic vitamin A, primarily in the forms of retinyl acetate, in pet food has become a standard practice to ensure optimal bioavailability and meet the requirements for this essential vitamin [23]. Consequently, the chemical synthesis of retinol has played a crucial role in nearly eliminating the risk of hypovitaminosis A in livestock, poultry, and pet nutrition, making a substantial contribution to the overall well-being of domesticated animals.

In recent years, research on vitamin A in pet nutrition has expanded beyond basic requirements to explore potential therapeutic applications. The investigations into the vitamin’s role in supporting the immune function, reducing inflammation, and promoting overall well-being have opened new avenues for enhancing the health of companion animals [36,37].

Studies in mammals have delved into the impact of vitamin A supplementation in managing specific health conditions, including dermatological issues, ocular disorders, and immune-mediated diseases [6,38,39,40,41]. 

## 3. Digestion and Metabolism of Vitamin A 

**Figure 2:**
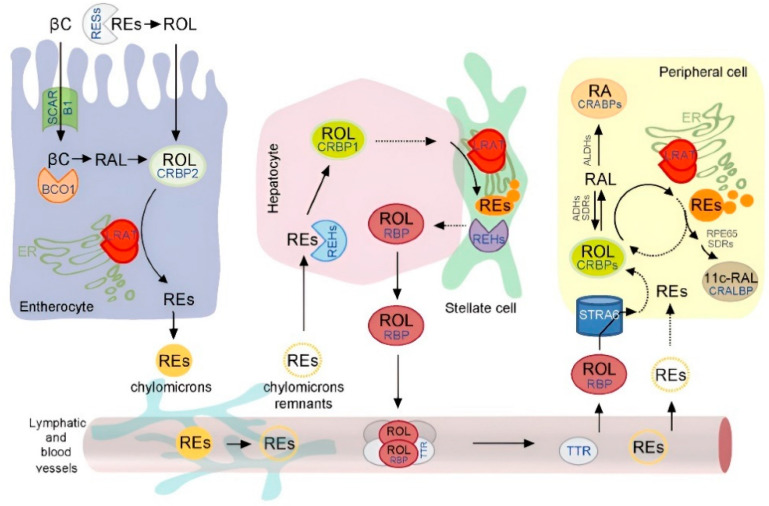
Diagram of the current model of vitamin A absorption, transport, and storage. Retinoid metabolism can be classified into three major processes—intestinal uptake, hepatic storage, and tissue-specific metabolism that are interconnected via lymphatic and blood vitamin A transport [47]. “Although each of these steps is characterized by a set of specialized proteins, lecithin:retinol acyltransferase (LRAT) plays a pivotal role in each of them. The abbreviations used are the following: 11c-RAL, 11-*cis*-retinal; ADHs, alcohol dehydrogenases; ALDHs, aldehyde dehydrogenases; βC, β,β-carotene; BCO1, β,β-carotene-15,15-dioxygenase; CRABPs, cellular retinoic acid-binding proteins; CRALBP, cellular retinaldehyde-binding protein; CRBP1, cellular retinol-binding protein 1; CRBP2, cellular retinol-binding protein 2; ER, endoplasmic reticulum; LRAT, lecithin:retinol acyltransferase; RA, all-*trans*-retinoic acid; RAL, all-*trans*-retinaldehyde; REHs, retinyl ester hydrolases; REs, retinyl esters; RESs, retinyl esterases; RPE65, retinoid isomerase; ROL, all-*trans*-retinol; RBP, serum retinol-binding protein; SCARB1, scavenger receptor class B, type I; SDRs, short-chain dehydrogenases/reductases; STRA6, stimulated by retinoic acid 6; TTR, transthyretin”.

The metabolic process of vitamin A in carnivores entails a sophisticated and multi-phase mechanism involving various enzymes and pathways. Initially present as retinyl esters, such as retinyl acetate, vitamin A undergoes hydrolysis facilitated by pancreatic lipases in the small intestine [5,42]. This enzymatic activity liberates retinol, which then forms mixed micelles upon combining with other lipids and bile salts, thus promoting efficient absorption by enterocytes ([43]; Figure 2). In carnivorous animals, the absorption of preformed vitamin A primarily takes place in the small intestine, particularly the jejunum [44,45]. Within this section, the preformed vitamin A is integrated into chylomicrons, lipid-containing particles, and transported via the lymphatic system to the liver [46].

In the liver, vitamin A is stored as retinyl esters in the hepatic stellate cells of dogs and cats, available for mobilization as required [7,10,48,49]. Conversely, provitamin A carotenoids, such as β-carotene found in plant-based sources, have the potential to undergo enzymatic cleavage in the intestinal mucosa, converting into retinaldehyde and retinol in canines [43]. However, the efficiency of this conversion process varies considerably; notably, dogs, akin to many other obligate carnivores, exhibit a limited ability to convert carotenoids compared to omnivores and herbivores [15]. Substantial amounts of intact β-carotene have been observed in the bloodstream of dogs following dietary supplementation, indicating a constrained conversion within the enterocytes [50,51].

Moreover, cats may be among the least efficient converters of β-carotene to vitamin A among domesticated animals. Previously, it was believed that felines were unable to convert β-carotene to vitamin A [52,53,54]. However, recent evidence suggests that cats can convert β-carotene into retinol, albeit to a very limited extent [55,56]. Consequently, the type of diet and dietary vitamin A supplementation emerge as critical factors in maintaining optimal vitamin A levels in both domesticated canines and felines.

Ancestral vertebrates underwent a loss of biosynthetic pathways for most vitamins, prompting the evolution of specialized mechanisms, such as dedicated transport proteins [57]. These proteins facilitate the absorption of dietary vitamins from the intestine, storage tissues, and serum [58]. In instances where there is a demand for vitamin A, the stored retinyl esters in the liver are mobilized and subsequently hydrolyzed to retinol. This retinol is then transported to target tissues predominantly via the retinol-binding protein (RBP) in most mammals [59,60,61]. The RBP plays a crucial role in regulating vitamin A homeostasis, ensuring its proper distribution and delivery to target tissues while preventing potential toxicity [5,62]. Interestingly, cats and dogs exhibit a lower dependence on the RBP for transporting vitamin A in plasma compared to other mammals. Instead, they primarily transport vitamin A as retinyl esters (mostly retinyl stearate, with lesser amounts of retinyl oleate and palmitate) bound to low-density lipoprotein (LDL) and very low-density lipoprotein (VLDL), in concentrations significantly higher (10–50 times) than other mammals ([6,63,64]; Table 1). The significance of this circulating pool of retinyl esters and its impact on the tissue metabolism of vitamin A remains unclear [65]. In dogs, cats, and other carnivores, only a portion of the retinol in the blood plasma is bound to the RBP, with an average ratio of retinol to total retinyl esters ranging from 0.2 to 1 to 1:1 [64,66,67]. This discrepancy underscores the necessity for species-specific investigations to comprehend the metabolism of vitamin A. It is of note that, in addition to potential biliary excretion, carnivores such as dogs and cats have a unique ability to eliminate excess vitamin A from their bodies through urine, specifically in the form of protein-bound vitamin A and retinyl esters [67].

Upon entering a cell, the retinol binds to the intracellular retinol-binding protein (CRBP) within the cytoplasm in both dogs and cats [76,77]. The CRBP acts as a carrier protein, facilitating the intracellular transport of the retinol to specific cellular compartments where it is utilized [78]. Within the cell, the retinol undergoes various metabolic transformations to fulfill the cell’s specific requirements.

A critical pathway involves the oxidation of retinol to retinaldehyde, a crucial precursor for the synthesis of the active forms of vitamin A. In mammals, including felines and canines, this oxidation reaction is mediated by enzymes called alcohol dehydrogenases (ADHs), particularly class I ADH and class IV ADH, which convert retinol to retinaldehyde [79,80,81,82,83,84,85]. Retinaldehyde can further metabolize to produce ATRA through the enzymatic activity of retinaldehyde dehydrogenases (RALDHs) [86,87,88]. The primary isoform responsible for this conversion, as evidenced by studies in dogs, is RALDH1A2 [89].

ATRA, the biologically active derivative of vitamin A, exerts its regulatory influence on the cellular metabolism in animals by selectively binding to nuclear receptors, specifically retinoic acid receptors (RAR) and retinoid X receptors (RXR). These receptors demonstrate widespread expression across various tissues, including the liver, kidney, intestine, adipose tissue, and immune cells [90,91,92]. Upon binding, the resultant retinoic acid-receptor complex interacts with specific DNA sequences, thereby modulating the gene expression in numerous biological processes [93,94,95].

## 4. Physiological Roles of Vitamin A 

Exploring the intricate mechanisms of vitamin A reveals its profound physiological significance. From visual perception to cellular proliferation, retinol and its active derivatives play multifaceted roles in sustaining bodily functions [5]. In this section, we delve into the dynamic interplay of vitamin A within the intricate landscape of canine and feline physiology.

### 4.1. Vision

**Figure 3:**
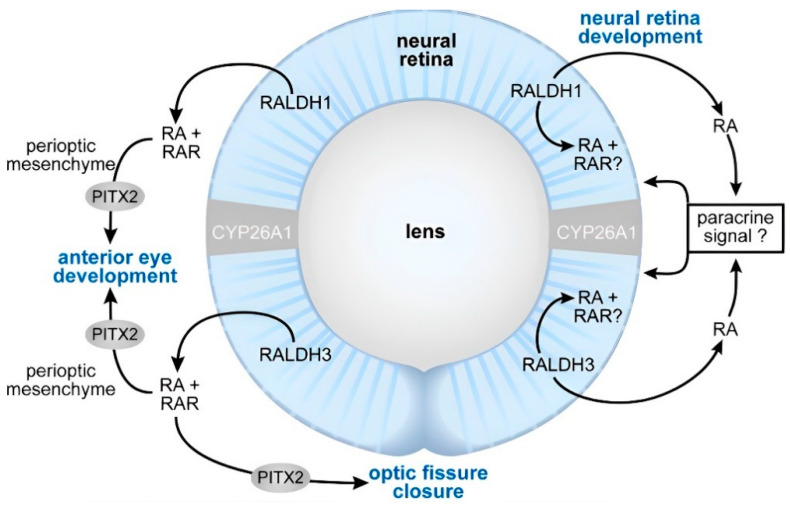
Schematic showing the proposed sites of RA (all-trans-retinoic acid; ATRA) function during eye morphogenesis (left) and differentiation (right) [102]. At the early stages of the eye development, the RA generated by RALDH1 and RALDH3 acts as a paracrine signal binding to the RARs located in the perioptic mesenchyme to support the anterior eye segment development and the closure of the optic fissure. Pitx2 is a RA/RAR-regulated transcription factor that is required both for anterior eye segment morphogenesis, as well as the closure of the optic fissure. At the later stages of development, the RA promotes the differentiation of the neural retina. The mechanism is unclear but could involve either a paracrine effect of the RA outside of the neural retina or a direct effect on the cells within the retina itself. https://creativecommons.org/licenses/by/3.0/ (accessed on 3 January 2024).

One of the most well-established functions of vitamin A in pets is its indispensable role in vision [11]. It plays a significant role in eye morphogenesis (Figure 3), as well as in the structure and function of the photoreceptor cells within the retina. In dogs and cats, the visual cycle comprises a complex biochemical process that ensures uninterrupted vision across varying light conditions. It initiates with the absorption of light by the photoreceptor cells in the retina, specifically the rod and cone cells [96,97,98]. These cells harbor visual pigments, including rhodopsin, a G-protein-coupled receptor, in rod cells, and iodopsins, a photopsin, in cone cells, comprised of opsin protein and light-sensitive retinal molecule [99,100,101].

After light absorption, the retinal shifts from its active 11-cis-retinal form to the inactive all-trans-retinal [103]. To maintain vision, the all-trans-retinal must convert back to 11-cis-retinal [104], a process occurring in the retinal pigment epithelium (RPE) behind the retina’s photoreceptor cells [103,105,106]. In the visual cycle, the all-trans-retinal is reduced to all-trans retinol, then oxidized to 11-cis retinal within the RPE cells [103,107]. This regenerated 11-cis-retinal is transported back to the photoreceptor cells, where it combines with opsin to create functional visual pigments [108]. The continuous regeneration of 11-cis-retinal is vital for maintaining light sensitivity and adapting to changes in illumination [109].

Furthermore, vitamin A is imperative for maintaining the structural integrity of the eye. Adequate vitamin A levels support the differentiation and upkeep of ocular tissues such as the cornea and conjunctiva [7,110].

### 4.2. Immune Function 

Vitamin A, acting through its metabolite ATRA, plays a critical role in regulating the immune system’s functionality in mammals. ATRA engages with the specific receptors known as RARs present within the immune cells, thereby initiating gene transcription processes [111,112]. This transcriptional activity leads to the synthesis of key proteins, such as Interferon Regulatory Factors (IRFs), which are essential for the proper function of immune cells [113,114,115,116]. These proteins contribute to regulating critical cellular processes including the differentiation, proliferation, and modulation of immune responses [117]. Ultimately, the influence of vitamin A on gene expression dynamics helps maintain a balanced and robust immune system, thereby enhancing the body’s ability to combat infections and sustain overall health [118].

**Figure 4:**
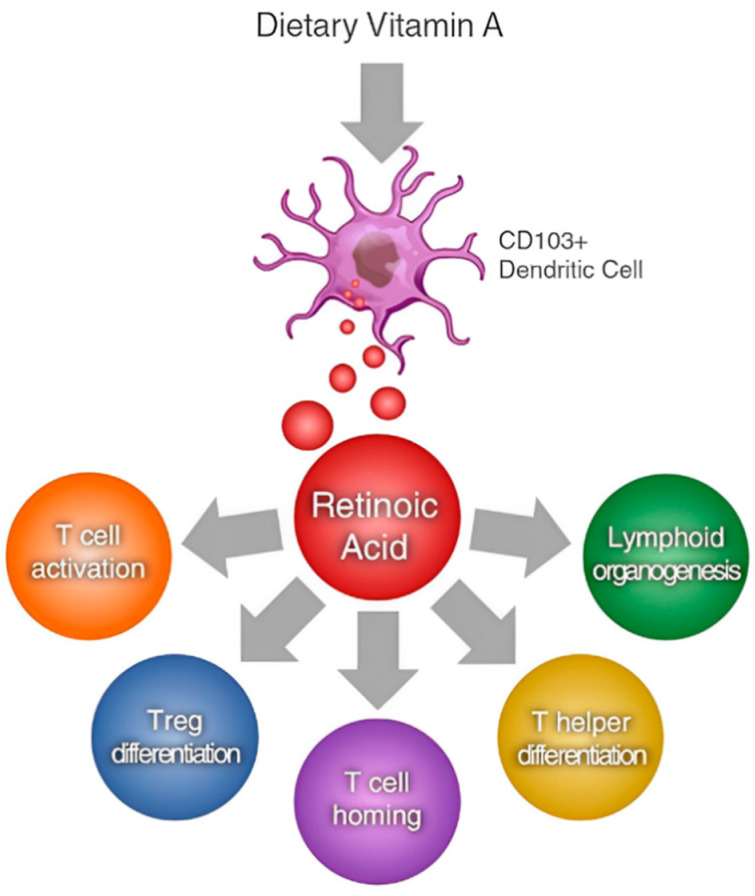
ATRA (all-trans-retinoic acid) as a modulator of T cell immunity [120].

Retinol and its derivatives are crucial for preserving the integrity of the mucosal surfaces found in the respiratory, gastrointestinal, and urogenital tracts, thereby acting as a protective barrier against pathogens [39]. Vitamin A plays a pivotal role in governing the differentiation and proliferation of various immune cells, including T and B lymphocytes, as well as the antigen-presenting cells (APCs) like dendritic cells ([40]; Figure 4). Due to its essential function in immune modulation, vitamin A is often referred to as “the anti-infective vitamin” [5,119]. As early as 1926, Mellanby [18], based on a comprehensive review of 330 post-mortem examinations of dogs, observed a significant correlation between bronchopneumonia, resulting from bacterial lung infections, and vitamin A deficiency, indicating a higher incidence among dogs with insufficient retinol levels.

Currently, retinol stands as one of the most extensively studied micronutrients concerning the immune function [121,122]. Moreover, vitamin A enhances mucosal immunity by stimulating the production of secretory IgA, IgM, and IgG antibodies [123,124], which are particularly crucial for defending against the pathogens at mucosal surfaces such as those in the gastrointestinal and respiratory tracts.

### 4.3. Growth, Cellular Differentiation, Morphogenesis, and Reproductive Health 

ATRA is recognized for its pivotal involvement in fundamental cellular processes such as growth (including in bones), differentiation, and organogenesis [125,126]. Through its specific binding to the RARs located in target cells, ATRA operates as a transcriptional regulator, modulating the expression of genes crucial for cell differentiation, proliferation, morphogenesis, and tissue development [127,128,129]. This regulatory mechanism orchestrates the intricate process of cell specialization and the formation of distinct tissues and organs during embryonic development [130].

With regards to cellular proliferation and differentiation, ATRA exerts its influence by promoting the progression of cells through the cell cycle, particularly the G1 phase. This is achieved through the activation of genes supportive of cell division while concurrently suppressing genes associated with cell cycle arrest [131]. Moreover, ATRA signaling contributes significantly to morphogenesis. For instance, it plays a critical role in organizing the trunk through three key morphogenetic processes: mesoderm segmentation (including somite size and bilateral registration), axial elongation (posterior extension), and the establishment of anterior–posterior identity within individual segments (regionalization) [132].

Vitamin A and its metabolites are recognized as vital for the optimal reproductive health and development of pets [10,133,134]. In the ovaries, ATRA exerts significant effects on granulosa cell functions such as proliferation, differentiation, and steroidogenesis, thereby contributing to follicular development [135,136,137,138]. Furthermore, ATRA is implicated in the regulation of gene expression related to reproduction and enhances estrogen production, particularly estradiol (E2), which is crucial for the menstrual cycle [139,140].

Moreover, ATRA is known to modulate uterine receptivity and facilitate embryo implantation by promoting the secretion of uterine factors necessary for embryo attachment and placental development [141,142]. Additionally, ATRA plays a pivotal role in male reproduction, influencing processes such as spermatogenesis, testicular development, and sperm production and motility [143,144].

### 4.4. Antioxidant Properties

Oxidative damage to DNA, proteins, and lipids is widely recognized as a significant factor contributing to aging and the development of various chronic diseases in cats and dogs [145]. Vitamin A plays a vital role as a systemic antioxidant, impacting various biological processes in animals, including pets [4,146]. Retinol possesses direct antioxidant properties due to its hydrophobic polyene chains, allowing it to quench singlet oxygen and neutralize radicals [147]. However, in high oxygen levels, vitamin A can undergo auto-oxidation, though it remains effective at physiological oxygen tensions [148,149].

Palace et al. [150] elucidated how retinol acts as a chain-breaking antioxidant, halting lipid peroxidation by reacting with peroxyl radicals, thus preventing the spread of lipid peroxidation in cells and the formation of hydroperoxides. Retinol efficiently scavenges peroxyl radicals in various lipid models, such as liposomes mimicking cell membranes. Additionally, ATRA, a vitamin A metabolite, serves as a potent transcriptional regulator, influencing the expression of genes related to antioxidant processes [151]. Specifically, ATRA upregulates genes involved in the glutathione (GSH) metabolism, bolstering cellular antioxidant defenses [152,153,154]. It also enhances superoxide dismutase (SOD) activity, crucial for neutralizing superoxide radicals [155]. ATRA modulates the oxidative stress pathways by downregulating the NADPH oxidase genes, thereby reducing reactive oxygen species production [156].

**Figure 5:**
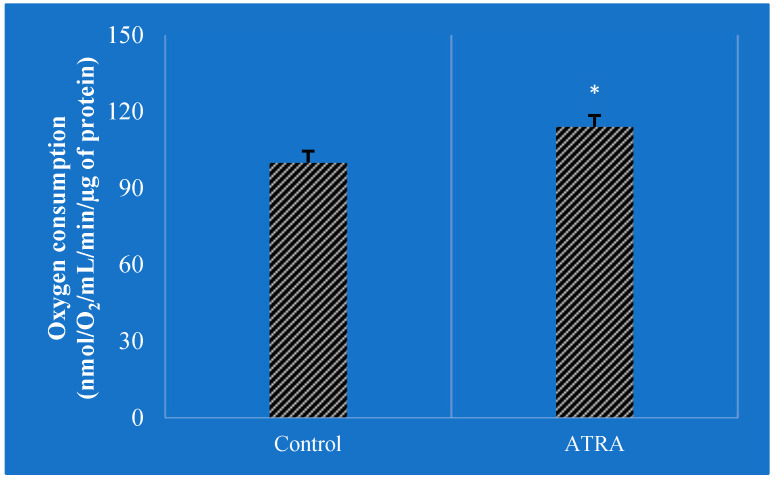
Oxygen consumption in adipocytes exposed to 2 µM of ATRA (all-trans-retinoic acid) was measured using Clarke’s electrode (adapted from Tourniaire et al. [157]). Control refers to control cells, which received the vehicle (dimethyl sulfoxide). Data are the mean  ±  SEM of three independent cultures per treatment condition. The assessment compared ATRA-treated cells to untreated cells and measured their oxygen consumption rates to determine if ATRA-induced gene expression changes altered cellular metabolism. ATRA increased oxygen consumption by 15% (* *p*  <  0.05).

Furthermore, ATRA enhances mitochondrial antioxidant activity by upregulating genes associated with biogenesis and defense mechanisms [157,158]. Specifically, Tourniaire et al. [157] found that ATRA increases the expression of genes linked to these processes, resulting in a heightened oxidative phosphorylation capacity and mitochondrial content (Figure 5). These findings suggest significant implications for managing oxidative stress. Additionally, ATRA induces autophagy, assisting cellular survival under oxidative stress conditions [159,160]. This process involves autophagosome acidification through a pathway independent of classic nuclear retinoid receptors, ultimately contributing to cellular homeostasis regulation [159].

## 5. Vitamin A Deficiency and Excess in Pets

Vitamin A is a fundamental micronutrient for pets, but, like many essential substances, it must be carefully regulated to avoid health complications. Both the deficiency and excess of vitamin A can have notable effects on pets’ well-being, necessitating an understanding of the causes, symptoms, and management of these conditions [161,162].

### 5.1. Vitamin A Deficiency

Vitamin A deficiency can arise in pets due to various factors, encompassing inadequate dietary intake, compromised absorption, or increased metabolic demands [146,163,164]. Pets consuming diets deficient in vitamin A or experiencing gastrointestinal disorders such as intestinal fat malabsorption are particularly susceptible. Furthermore, certain medical conditions or periods of rapid growth may elevate the requirement for vitamin A in dogs and cats [35,165].

One of the primary physiological roles of vitamin A involves maintaining the optimal visual function. Consequently, the felines and canines deficient in this vitamin may manifest symptoms such as nyctalopia, reduced vision in low light, conjunctivitis, xerosis with keratitis and corneal neovascularization, photophobia, mydriasis in normal lighting, delayed pupillary light reflex, progressive retinal cell degeneration, cataract formation, or even complete blindness in severe cases [19,27,146]. Additionally, common neurological manifestations of progressive vitamin A deficiency include an altered mental state, seizures, nystagmus, ataxia, kyphosis, hyperesthesia, muscle wasting, nerve degeneration, and impaired nerve conduction [32,146,166].

Hypovitaminosis A in pets also frequently leads to weight loss, bronchial epithelial metaplasia, squamous metaplasia in the salivary glands and endometrium, a dry and lackluster coat, dermatological issues, and a compromised immune function predisposed to infections [18,32,33,119,167]. Reproductive complications such as infertility or dystocia can also arise due to vitamin A deficiency in breeding animals [15,168].

The diagnosis of vitamin A deficiency in pets typically involves a comprehensive evaluation including clinical signs, dietary history, and laboratory analyses. Blood assays may reveal diminished vitamin A levels, though these findings can be inconclusive due to various factors such as the presence of different vitamin A forms in plasma, carnivores’ ability to renally excrete vitamin A, individual variabilities, age, physiological and nutritional influences, diurnal fluctuations, and sampling stress, among others [64,66,169,170,171,172,173,174,175,176]. Furthermore, it is important to recognize that veterinary practitioners often have a limited availability of the specialized instrumentation required for measuring the vitamin A levels in various tissues. Finally, veterinarians may also scrutinize the pet’s diet and overall health status to elucidate the root cause of the deficiency [9]. 

The treatment of vitamin A deficiency in dogs and cats involves administering vitamin A supplements orally or via injections, contingent upon the severity of the condition [6,9]. Additionally, transitioning to commercial pet foods formulated to meet nutritional requirements may be warranted if the pet was previously fed deficient homemade diets. The regular monitoring of the pet’s response and addressing any underlying health issues contributing to the deficiency are imperative for successful management [177,178]. According to Silver [6], the following examples of diseases often linked to retinol deficiency in dogs and cats can be mitigated or treated with supplements of retinoids:


✓Retinoid-responsive dermatoses;

✓Sebaceous gland disorder;

✓Canine icthyosis;

✓Solar dermatosis of dogs and cats;

✓Feline muzzle folliculitis;

✓Nyctalopia;

✓Xerophthalmia;

✓Keratoconjunctivitis sicca;

✓Squamos metaplasia;

✓Squamous cell carcinoma;

✓Epitheliotrophic T cell lymphoma;

✓Respiratory infections;

✓Intestinal inflammation;

✓Hyperkeratinization of the epithelial surfaces;

✓Seborrhea;

✓Keratoacanthoma;

✓Haircoat problems;

✓Schnauzer comedo syndrome;

✓Alopecia;

✓Increased susceptibility to infection;

✓Exfoliation;

✓Delayed wound healing;

✓Sebaceous adenitis;

✓Follicular dysplasia.


### 5.2. Vitamin A Excess

Hypervitaminosis A, or vitamin A overdose, can occur in canines and felines when they consume excessively high levels of vitamin A, either through their diet or via supplementation. Dogs and cats are at a higher risk of experiencing vitamin A overdose if they devour excessive amounts of raw liver from fish, swine, or cattle over extended periods, as it contains particularly high levels of this vitamin [68,161,179]. 

Generally, felines and canines exhibit a higher tolerance to developing hypervitaminosis A compared to other domesticated mammals and poultry [6]. This heightened tolerance can be attributed to several factors. Firstly, dogs and cats predominantly transport vitamin A as retinyl ester in their blood plasma [180]. Unlike in many mammals, elevated retinyl ester levels in plasma among carnivores do not correlate with the signs of vitamin A overdose or postprandial effects [64]. Secondly, carnivores eliminate excess vitamin A through urine in the form of protein-bound vitamin A and retinyl esters [67]. Dogs exhibit a higher urinary retinol excretion capacity compared to cats ([64,66]; Table 2), ranging, for example, from 15 to 63% of the intake in beagle dogs [180]. This efficient excretion mechanism prevents the accumulation of retinyl esters in the liver and kidney cells, leading to the manifestation of hypervitaminosis A only at extremely high intake levels of vitamin A (>90 mg of retinol/kg BW consumed over a prolonged period) [180]. While humans also excrete the metabolic products of retinol via urine [181], the urinary excretion of unmetabolized retinol or its esters in healthy non-carnivorous domesticated animals is unheard of. The ability of carnivores to rapidly excrete vitamin A and its esters via urine may be an evolutionary adaptation, allowing them to consume potentially high amounts of retinol in the wild (e.g., liver consumption).

The symptoms of hypervitaminosis A in pets vary depending on the severity and duration of the exposure. The studies investigating retinol overdose employed extraordinarily high doses of vitamin A, ranging from 3,500,000 to 15,000,000 IU per kg of diet, administered to cats consistently over a period of up to 10 months [48,182,183]. These doses exceeded the latest NASEM requirement estimate for adult cats by up to 4500 times. The dogs fed diets supplemented with 100,000 IU of vitamin A per 1000 kcal of diet for 44 weeks exhibited no signs of hypervitaminosis A or any adverse health effects [10]. 

The early signs of hypervitaminosis A often include gastrointestinal disturbances such as diarrhea and loss of appetite [183]. As the overdose progresses, dogs and cats may exhibit neurological symptoms such as lethargy, weakness, disorientation, and seizures, along with bone demineralization and reduced thyroxin levels in the blood plasma [68].

Preventing vitamin A overdose in pets entails feeding them a balanced diet formulated to meet their nutritional needs without excessive supplementation (Table 3). Pet owners should refrain from feeding large amounts of liver or pure vitamin A supplements without consulting a veterinarian. Regular veterinary check-ups can aid in identifying and addressing any nutritional imbalances before they adversely affect pet health.

## 6. Interactions with Other Micronutrients

Understanding vitamin A’s interactions with other micronutrients is vital for maintaining overall health and preventing deficiencies or overdose. Here, we explore the intricate relationships between vitamin A and several key micronutrients.

### 6.1. Vitamin A and Vitamin D

**Figure 6:**
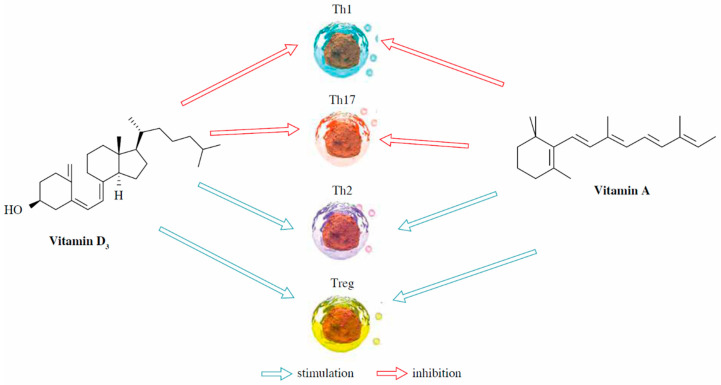
Immunomodulatory capacity of vitamin A and D [186]. Th = T helper cells: Th1 (they primarily produce cytokines such as interferon-gamma (IFN-γ) and interleukin-2 (IL-2) and are involved in cell-mediated immunity), Th17 (they produce cytokines such as interleukin-17 (IL-17) and interleukin-22 (IL-22) and are involved in the defense against extracellular pathogens), and Th2 (they produce cytokines such as interleukin-4 (IL-4), interleukin-5 (IL-5), and interleukin-13 (IL-13) and are involved in humoral immunity and allergic responses); and Treg = regulatory T cells (they express the transcription factor Foxp3 and play a crucial role in immune tolerance and regulation).

Vitamin A and vitamin D are integral to various physiological processes, including immune regulation, bone metabolism, and cellular differentiation [10,185,186]. The interplay between these vitamins is complex, involving their active forms binding to specific nuclear receptors. The active forms of vitamin A, such as ATRA and 9-cis retinoic acid (9-cis RA), interact with RAR and RXR, while 1,25-dihydroxyvitamin D_3_ (1,25(OH)_2_D_3_) binds to the vitamin D receptor (VDR) and RXR [187]. These receptors form heterodimers that bind to response elements like the vitamin D response element (VDRE) and the retinoic acid response element. The research indicates that 9-cis RA can modulate the effects of 1,25(OH)_2_D_3_, leading to diverse outcomes ranging from antagonistic to synergistic [187,188,189,190,191]. Notably, ATRA can influence the expression of the vitamin D-binding protein (DBP) complex, which is pivotal for the cellular uptake and actions of vitamin D, thus implicating vitamin A in the modulation of the vitamin D metabolism within specific cell types [187]. Moreover, vitamin A and vitamin D collaboratively regulate immune responses in the innate lymphoid cells (ILCs) ([186]; see Figure 6). However, ATRA and 1,25(OH)_2_D_3_ may also exert antagonistic effects on the expression of effector cytokines and gut-homing integrin by mammalian ILCs. The balance between these vitamins could be a key determinant in ILC activity and associated diseases, including allergic inflammation [189].

### 6.2. Vitamin A and Vitamin E

Vitamin A and vitamin E are both powerful antioxidants that protect cells from oxidative damage and play essential roles in maintaining skin health, vision, and the immune function [4,192]. While they have distinct antioxidant properties, they may also interact synergistically to enhance each other’s effectiveness. In experiments conducted within a unilamellar liposomal system comprised of phosphatidylcholine, there is evidence indicating that α-tocopherol enhances the antioxidant efficacy of all-trans-retinol by reducing its autooxidation [193]. This action likely occurs through the concerted scavenging of radicals, leading to the synergistic protection of the lipid system against peroxidative stress [194]. Consequently, this mechanism could potentially reduce the consumption of α-tocopherol over time. However, given that retinol exhibits relatively weak direct antioxidant properties, further investigations are warranted to accurately quantify the potential interaction between these vitamins in vivo. Moreover, vitamin E aids in the body’s utilization of vitamin A while also regulating vitamin A stores, thereby mitigating the risk of hypervitaminosis A. Nevertheless, the excessive intake of vitamin E may negatively influence the vitamin A storage levels in the body [6].

### 6.3. Vitamin A and C

Vitamin C is synthesized endogenously within the liver across a variety of species, including canines and felines, and is distributed widely throughout the body’s [195]. This essential micronutrient serves pivotal physiological functions in various metabolic pathways, including tissue growth and maintenance, the mitigation of oxidative stress, and the modulation of the immune system [1].

Administering vitamin A and C, either prior to or following the induction of stress in mammals, has been shown to significantly enhance the activities of crucial antioxidant enzymes such as SOD, glutathione-S-transferase, and catalase [196]. Moreover, there is a notable increase in the levels of reduced glutathione, accompanied by a decrease in lipid peroxidation. Importantly, this combined treatment demonstrates markedly superior outcomes compared to the use of either vitamin alone. Similarly, Hosseini Omshi et al. [197] reported that the supplementation of vitamins A and C in animals may offer promising effects against the imbalance between oxidants and antioxidants.

### 6.4. Vitamin A and Zinc

Zinc, an indispensable trace element, plays a pivotal role in numerous enzymatic reactions, immune modulation, and the process of wound healing [198]. Studies in humans and chickens, as well as ex vivo research using cultures of spleen leukocytes from dogs, have suggested the existence of a synergistic relationship between the dietary intake of zinc and the metabolism and status of vitamin A [199,200,201]. Simultaneous long-term zinc and vitamin A supplementation was shown to be associated with reduced parasitic gastrointestinal infections caused by Giardia lamblia and Ascaris lumbricoides in humans [202]. The influence of zinc extends to various facets of vitamin A metabolism, encompassing absorption, transportation, and utilization. This influence arises from the regulatory function of zinc in vitamin A transportation, primarily facilitated through the synthesis of retinol-binding protein (RBP) in the liver. Additionally, zinc serves as a crucial cofactor in the synthesis of enzymes that regulate the absorption and function of vitamin A [203]. Furthermore, zinc plays a pivotal role in the oxidative conversion of retinol to retinal, necessitating the action of zinc-dependent retinol dehydrogenase enzymes [204]. Studies indicate that in animals a deficiency in zinc consistently leads to reduced plasma vitamin A concentrations, despite a diet being nutritionally adequate in vitamin A [203]. Moreover, zinc deficiency has been shown to diminish the lymphatic absorption of retinol in animal models, a phenomenon linked to a decline in the output of lymphatic phospholipids due to compromised biliary secretion into the intestinal lumen [205]. Conversely, vitamin A is required for the absorption and utilization of zinc by improving intestinal functionality [201,204]. 

## 7. Comparing Vitamin A Requirements: Livestock vs. Pets

Variability in the requirements for vitamin A is evident across different species and even among individuals within the same species [5,7,62,206,207]. Understanding these variations is crucial for maintaining the optimal health and well-being of animals under human care. Due to extensive artificial selection, dogs have evolved into one of the most morphologically diverse vertebrate species, encompassing approximately 400 distinct breeds [208,209]. Similarly, domestic cats exhibit a wide range of breed variations and morphological traits [210]. The selective breeding of dogs and cats has primarily been motivated by considerations of aesthetics, morphology, and behavior [211].

In contrast to companion animals, modern livestock species such as swine, poultry, and cattle have undergone intense selection and breeding for traits such as rapid growth, lean meat deposition, efficient feed conversion, and high egg and milk production [5,62,206,207]. This genetic manipulation imposes additional metabolic demands and alters the requirement for essential nutrients, including vitamins. For instance, modern pig breeds exhibit an increased metabolic turnover and oxidative stress levels akin to those of endurance athletes [212]. Consequently, the demand for fat-soluble vitamins in livestock has increased significantly over the past few decades [5,62,206,207,213].

While certain dog breeds may be exceptions due to selective breeding for enhanced performance [214], the overall trend suggests that the vitamin A requirements for companion animals, particularly dogs and cats, may not have escalated to the same extent as those of livestock species. This discrepancy underscores a fundamental difference between the breeding pressures shaping the nutrient needs of pets versus those of livestock. 

The specific vitamin A requirements for domesticated animals depend on various factors, including the animal’s age, weight, and reproductive status, as well as other parameters [5]. For example, pregnant and lactating pets have increased vitamin A requirements to support fetal development and milk production [35,215]. Similarly, young, growing animals require higher levels of vitamin A to support bone development and overall growth [216]. In contrast, mature animals may have lower vitamin A requirements but still need adequate amounts to maintain health and the immune function. We propose that the ideal dosage of vitamin supplementation in companion animals depends on promoting optimal growth and development, as well as achieving a balance marked by maximal physiological well-being, including immune competence, while ensuring adequate bodily reserves are maintained. Table 4 presents the minimum requirements and practical dosage recommendations for vitamin A in cats and dogs sourced from various references. The authors have published similar information for poultry, swine, and cattle elsewhere. For comparison with felines and canines, readers are referred to these peer-reviewed data [62,206,207]. Furthermore, it should be noted that NASEM estimates for vitamins represent the minimum levels necessary to prevent clinical deficiencies and may not necessarily ensure optimal health, including the immune function, and sufficient bodily reserves [217,218].

Overall, while the vitamin A requirements of livestock and pets share similarities, such as the need for adequate levels to support growth, development, and overall health, significant differences between the two arise due to variations in the goals of genetic selection.

## 8. Future Directions and Research Gaps

As the field of companion animal wellness progresses, there are numerous avenues for future research aimed at understanding the role of vitamin A in the health and well-being of pets. In this section, we outline potential directions for future investigation and emphasize key research gaps that require attention within the scientific community.

Although there have been notable advancements in elucidating the metabolism of vitamin A in humans and laboratory animals, our comprehension of these mechanisms in companion animals, such as dogs and cats, remains comparatively limited. This disparity in knowledge presents obstacles in effectively optimizing the dietary management of pets. To bridge this gap, it is proposed to widen usage of in vitro simulation techniques and ex vivo methodologies to investigate the metabolism of vitamin A in companion animals [220]. This approach is advocated primarily due to ethical considerations.

Furthermore, investigating rodents as potential models for elucidating the vitamin A metabolism in dogs and cats should be assessed [221,222,223]. This implies that the translational-related challenges between the models regarding vitamin A need clarification. By directing the research efforts towards uncovering the specific pathways involved in the absorption, storage, and utilization of vitamin A, potentially valuable insights can be gained. If proven to be applicable, these insights might facilitate the customization of dietary vitamin A levels to suit different breeds of dogs and cats, thereby enhancing overall pet health and wellness.

Genetic factors play a crucial role in determining the vitamin A requirements and response to supplementation [224,225]. Investigating genetic variability among different pet breeds and its influence on the metabolism and function of vitamin A could aid in tailoring personalized nutrition strategies for optimal health outcomes. Integrating genomic approaches with nutritional studies will pave the way for precision nutrition in companion animals. Nevertheless, genetic association studies remain challenging, even in human populations. Recognizing the potential challenges for similar studies in pets would provide further confidence.

Long-term studies tracking vitamin A status and its correlation with various health parameters in pets are essential for establishing causal relationships and understanding the impact of vitamin A deficiency or excess on overall health outcomes [226,227]. Longitudinal research designs, encompassing diverse populations of pets across different life stages and environmental conditions, will provide robust evidence to guide nutritional recommendations and preventive healthcare strategies.

Vitamin A is known to play a critical role in modulating the immune function and reducing the risk of infectious and chronic diseases in humans [176,228]. Similarly, investigating the immunomodulatory effects of vitamin A in pets can offer valuable insights into enhancing immune resilience and mitigating disease susceptibility. This includes reducing the incidence of respiratory infections and improving wound healing times. Specifically, exploring the translational potential of molecular studies on retinoids’ impact on the immune function in companion animals holds significant promise for animal health, a concept already supported by the research in humans [229]. Understanding how vitamin A enhances mucosal immunity, as evidenced by its stimulation of secretory IgA, IgM, and IgG antibodies [39,230], forms a crucial foundation for potential applications in veterinary practice for pets [231,232]. These antibodies play a pivotal role in defending mucosal surfaces, such as those in the gastrointestinal and respiratory tracts, against pathogens, making the findings a promising research direction for enhancing the health and well-being of companion animals [8].

Pets are frequently exposed to diverse pathogens in their environments, so enhancing their natural defenses through vitamin A interventions could lead to reduced disease susceptibility and improved overall health outcomes. Moreover, comprehending how retinoids influence gene expression related to immunity could provide insights into tailoring treatments for individual animals, thereby enhancing their well-being and quality of life [90,233]. Ultimately, the application of the molecular findings on retinoids in pets has the potential to transform veterinary medicine, offering more effective and personalized care for our beloved companions.

The stability and bioavailability of vitamin A from pet food and animal feed formulations can vary depending on factors such as the vitamin A formulation (from a commercial source), the ingredient composition, processing methods, and storage conditions [110,234,235,236,237]. Systematic assessments of vitamin A bioavailability in commercial pet foods are necessary to ensure adequate nutrient delivery and to optimize dietary formulations for pet health and wellness. Incorporating advanced analytical techniques and in vitro digestion models can facilitate the accurate determination of vitamin A bioavailability in pet food matrices.

## 9. Conclusions

The multifaceted role of vitamin A in the health and well-being of companion animals is undeniable. From its crucial involvement in vision, the immune function, and reproduction to its antioxidant properties, vitamin A serves as a cornerstone of optimal pet nutrition. Through historical perspectives, we have seen how our understanding of vitamin A’s importance has evolved, leading to the establishment of recommended dietary allowances and the development of commercial pet foods aimed at meeting the essential nutritional requirements.

The intricate processes of digestion, metabolism, and physiological functions elucidate the complexities of ensuring adequate vitamin A levels in pets, especially considering their unique dietary needs as obligate carnivores. While deficiencies can lead to a myriad of health issues, including impaired vision, a compromised immune function, and reproductive complications, excess intake may also carry potential concerns, underscoring the importance of balanced nutrition and careful supplementation.

Exploring interactions with other micronutrients further emphasizes the interconnectedness of dietary components in promoting overall health. Future research directions, including investigating genetic variability, long-term studies tracking vitamin A status, and exploring immunomodulatory effects, offer promising avenues for enhancing our understanding of vitamin A’s role in companion animal nutrition. Additionally, considerations of stability and bioavailability in pet food formulations underscore the importance of optimizing nutritional strategies to ensure the well-being of our beloved animal companions. By addressing these research gaps and advancing our knowledge, we can continue to improve the nutritional management of pets, ultimately enhancing their quality of life and longevity.

## Figures and Tables

**Table 1:** Blood plasma or serum concentrations of retinol and retinyl esters in dogs, cats, and humans.

Species	Retinol	Retinyl Stearate	Retinyl Palmitate + Oleate	Total Retinyl Esters	Reference
Dogs	0.3–1.0 mg/L	0.8–1.0 mg/L	0.5–0.7 mg/L	1.3–1.7 mg/L	[68] ^1^
	0.6–0.8 mg/L	0.9–0.1 mg/L	0.6–0.7 mg/L	1.5–1.7 mg/L	[69] ^1^
	0.9–1.3 mg/L	0.23–0.45 mg/L	0.3–0.4 mg/L	0.53–0.85 mg/L	[64] ^1^
	2.3–4.1 µmol/L	3.5–10.6 µmol/L	1.4–6.2 µmol/L	4.9–16.8 µmol/L	[70] ^1^
	2.7–3.4 µmol/L	Not measured	4.5–12.0 * µmol/L	-	[71] ^2^
	642 ng/mol	916 ng/mol	609 ng/mol	1525 ng/mol	[63] ^1^
Cats	0.2–1.6 mg/L	0.3–0.4 mg/L	0.1–0.2 mg/L	0.4–0.6 mg/L	[72] ^1^
	0.24 mg/L	0.4 mg/L	0.3 mg/L	0.7 mg/L	[66] ^1^
	366–533 nmol/L	247–327 nmol/L	162–203 nmol/L	409–530 nmol/L	[54] ^1^
	213 ng/mol	323 ng/mol	165 ng/mol	488 ng/mol	[63] ^1^
Humans	0.065–3.14 μmol/L	-	-	0.00–0.11 μmol/L	[73] ^2^
	2.0–4.0 μmol/L	-	-	0.1–0.2 μmol/L	[74] ^1^
	2.1–2.4 μmol/L	-	-	0.054–0.056 μmol/L	[75] ^2^

* Only retinyl palmitate was measured; ^1^ blood plasma; and ^2^ blood serum.

**Table 2:** Retinol and retinyl ester concentrations in the urine of domestic cats and dogs (mean ± SD).

Species	Total Vitamin A	Retinol	Retinyl Stearate	Retinyl Palmitate/Oleate	Reference
Dogs	0.44 ± 0.55 µg/mL	0.22 ± 0.04 µg/mL	0.006 ± 0.008 µg/mL	0.21 ± 0.39 µg/mL	[64]
	0.6 ± 0.4 mg/L	Not indicated	Not indicated	Not indicated	[180]
	580 ng/mL	Not measured	Not measured	Not measured	[54]
Cats	22 ± 21.0 ng/mL	Not detected	15 ± 13.6 ng/mL	9 ± 7.6 ng/mL/11 ± 17.5 ng/mL	[66]
	131 µg/dL	Not indicated	Not indicated	Not indicated	[180]

**Table 3:** Upper safe levels of vitamin A in dogs and cats as provided by the various literature sources.

Source	Dogs	Cats
[183]	33,330 IU/kg of diet	100,000 IU/kg of diet
[11]	Puppies and breeding bitches: 50,000 IU/kg of diet Adult: 210,000 IU/kg of diet	330,000 IU/kg of diet
[10]	100,000 IU/1000 kcal of diet	-
[184]	250,000 IU/kg of diet (DM basis)	333,300 IU/kg of diet (DM basis)
[35]	400,000 IU/kg of diet (DM basis)	Adult and growth: 400,000 IU/kg of diet (DM basis) Reproduction: 333,333 IU/kg of diet (DM basis)

**Table 4:** Minimum requirement estimates (NASEM), minimum recommended levels (FEDIAF), minimum levels (AAFCO), and recommendations for optimum supply (AWT) of vitamin A in dog and cat food (IU/kg of diet; dry matter basis).

Source	Dogs			Cats		
	Growing	Reproduction	Adult	Growing	Reproduction	Adult
[33]	-	-	-	3333 *	5999 *	-
[32]	3336 *	-	-	-	-	-
[11]	3533 *	3533 *	3533 *	3333 *	6666 *	3333 *
[35]	5000 *	5000 *	6060–7020 *	3333–4444 *	9000 *	9000 *
[184]	5000 *	5000 *	5000 *	6668 *	6668 *	3332 *
[219]	8000–12,000 **	8000–12,000 **	8000–12,000 **	15,000–25,000 **	15,000–25,000 **	15,000–25,000 **

* Minimum; and ** Recommendations for optimum supply.

## Data Availability

Not applicable.

## Abbreviations

SOD	Superoxide dismutase
1,25(OH)2D3	1,25-dihydroxyvitamin D3
11c-RAL	11-cis-retinal; ADHs, alcohol dehydrogenases
ADHs	Alcohol dehydrogenases
ALDHs	Aldehyde dehydrogenases
APCs	Antigen-presenting cells
ATRA	All-trans retinoic acid
BCO1	β,β-carotene-15,15-dioxygenase
CRABPs	Cellular retinoic acid-binding proteins
CRALBP	Cellular retinaldehyde-binding protein
CRBP	Intracellular retinol-binding protein
CRBP1	Cellular retinol-binding protein 1
CRBP2	Cellular retinol-binding protein 2
DBP	Vitamin D-binding protein
DNA	Deoxyribonucleic acid
E2	Estradiol
ER	Endoplasmic reticulum
GSH	Glutathione
Ig	Immunoglobulin
ILCs	Innate lymphoid cells
IRFs	Interferon Regulatory Factors
LDL	Low-density lipoprotein
LRAT	Lecithin:retinol acyltransferase
NADPH	Nicotinamide adenine dinucleotide phosphate
Pitx2 i	RA/RAR-regulated transcription factor
RA	All-trans-retinoic acid
RAL	All-trans-retinaldehyde
RALDHs	Retinaldehyde dehydrogenases
RAR	Retinoic acid receptors
RBP	Serum retinol-binding protein
REHs	Retinyl ester hydrolases
REs	Retinyl esters
RESs	Retinyl esterases
ROL	All-trans-retinol
RPE	Retinal pigment epithelium
RPE65	Retinoid isomerase
RXR	Retinoid X receptors
SCARB1	Scavenger receptor class B, type I
SDRs	Short-chain dehydrogenases/reductases
STRA6	Retinoic acid 6
Th	T helper cells
Treg	regulatory T cells
TTR	Transthyretin
VDR	Vitamin D receptor
VDRE	Vitamin D response element
VLDL	Very low-density lipoprotein
βC	β,β-carotene

## References

[1] Gordon D.S., Rudinsky A.J., Guillaumin J., Parker V.J., Creighton K.J. (2020). Vitamin C in Health and Disease: A Companion Animal Focus. Top. Companion Anim. Med..

[2] Stockman J., Villaverde C., Corbee R.J. (2021). Calcium, Phosphorus, and Vitamin D in Dogs and Cats: Beyond the Bones. Vet. Clin. N. Am. Small Anim. Pract..

[3] Fan Z., Bian Z., Huang H., Liu T., Ren R., Chen X., Zhang X., Wang Y., Deng B., Zhang L. (2023). Dietary Strategies for Relieving Stress in Pet Dogs and Cats. Antioxidants.

[4] Shastak Y., Gordillo A., Pelletier W. (2023). The relationship between vitamin A status and oxidative stress in animal production. J. Appl. Anim. Res..

[5] Shastak Y., Pelletier W. (2023). The role of vitamin A in non-ruminant immunology. Front. Anim. Sci..

[6] Silver R.J. Liquid A Drops. Technical Report for Veterinarian Use Only. VBS Direct Ltd. 1 Mill View Close, Bulkeley, Cheshire, SY14 8DB. 2024. https://vbsdirect.co.uk/files/Vitamin_A_Tech_Report2.pdf.

[7] Carazo A., Macáková K., Matoušová K., Krčmová L.K., Protti M., Mladěnka P. (2021). Vitamin A Update: Forms, Sources, Kinetics, Detection, Function, Deficiency, Therapeutic Use and Toxicity. Nutrients.

[8] Shoveller A.K., De Godoy M.R., Larsen J., Flickinger E. (2016). Emerging Advancements in Canine and Feline Metabolism and Nutrition. Sci. World J..

[9] Watson T.D. (1998). Diet and skin disease in dogs and cats. J. Nutr..

[10] Morris P.J., Salt C., Raila J., Brenten T., Kohn B., Schweigert F.J., Zentek J. (2012). Safety evaluation of vitamin A in growing dogs. Br. J. Nutr..

[11] NASEM (The National Academies of Sciences, Engineering, and Medicine) (2006). Nutrient Requirements of Dogs and Cats.

[12] Di Cerbo A., Morales-Medina J.C., Palmieri B., Pezzuto F., Cocco R., Flores G., Iannitti T. (2017). Functional foods in pet nutrition: Focus on dogs and cats. Res. Vet. Sci..

[13] Domínguez-Oliva A., Mota-Rojas D., Semendric I., Whittaker A.L. (2023). The Impact of Vegan Diets on Indicators of Health in Dogs and Cats: A Systematic Review. Vet. Sci..

[14] Baker H., Schor S.M., Murphy B.D., DeAngelis B., Feingold S., Frank O. (1986). Blood vitamin and choline concentrations in healthy domestic cats, dogs, and horses. Am. J. Vet. Res..

[15] Green A.S., Fascetti A.J. (2016). Meeting the vitamin A requirement: The efficacy and importance of β-carotene in animal species. Sci. World J..

[16] Verbrugghe A., Dodd S. Plant-Based Diets for Dogs and Cats. World Small Animal Veterinary Association Congress Proceedings. 2019. https://www.vin.com/doc/?id=9382843.

[17] McCollum E.V., Davis M. (1913). The necessity of certain lipins in the diet during growth. J. Biol. Chem..

[18] Mellanby E. (1926). Diet and disease, with special reference to the teeth, lungs, and pre-natal feeding. Lancet.

[19] Stimson A.M., Hedley O.F. (1933). Observations on Vitamin A Deficiency in Dogs. Public Health Rep..

[20] Semba R.D. (1999). Vitamin A as “anti-infective” therapy, 1920–1940. J. Nutr..

[21] Leitner Z.A. (1951). The Clinical Significance Of Vitamin-A Deficiency. Br. Med. J..

[22] Lanska D.J. (2010). Chapter 29: Historical aspects of the major neurological vitamin deficiency disorders: Overview and fat-soluble vitamin A. Handbook of Clinical Neurology.

[23] Shastak Y., Pelletier W., Kuntz A. (2024). Insights into Analytical Precision: Understanding the Factors Influencing Accurate Vitamin A Determination in Various Samples. Analytica.

[24] Semba R.D. (2012). On the ‘Discovery’ of Vitamin A. Ann. Nutr. Metab..

[25] (1932). Isolation of Vitamin A. Nature.

[26] Holmes H.N., Corbet R.E. (1937). The Isolation of Crystalline Vitamin A1. J. Am. Chem. Soc..

[27] Scott P.P., Greaves J.P., Scott M.G. (1964). Nutritional blindness in the cat. Exp. Eye Res..

[28] NASEM (The National Academies of Sciences, Engineering, and Medicine) (1974). Nutrient Requirements of Dogs.

[29] NASEM (The National Academies of Sciences, Engineering, and Medicine) (1978). Nutrient Requirements of Cats.

[30] Case L.P., Daristotle L., Hayek M.G., Raasch M.F. (2011). Pet Foods in: Canine and Feline Nutrition.

[31] (2023). PFI (Pet Food Institute). https://www.petfoodinstitute.org/about-pet-food/nutrition/history-of-pet-food/.

[32] NASEM (The National Academies of Sciences, Engineering, and Medicine) (1985). Nutrient Requirements of Domestic Animals: Nutrient Requirement of Dog and Cats.

[33] NASEM (The National Academies of Sciences, Engineering, and Medicine) (1986). Nutrient Requirements of Domestic Animals: Nutrient Requirement of Cats.

[34] Dodd S.A.S., Shoveller A.K., Fascetti A.J., Yu Z.Z., Ma D.W.L., Verbrugghe A. (2021). A Comparison of Key Essential Nutrients in Commercial Plant-Based Pet Foods Sold in Canada to American and European Canine and Feline Dietary Recommendations. Animals.

[35] FEDIAF Scientific Advisory Board Nutritional Guidelines for Complete and Complementary Pet Food for Cats and Dogs. 2021. pp. 1–98. https://europeanpetfood.org/wp-content/uploads/2022/03/Updated-Nutritional-Guidelines.pdf.

[36] Kumar N., Gupta R.C., Nagarajan G. (2016). Vitamin A deficiency in dogs: A review. J. Anim. Res..

[37] Stasieniuk E., Barbi L., Bomcompagni T. Recent Advances in Nutritional Immunomodulation in Dogs and Cats. All Pet Food. 2022. https://en.allpetfood.net/entrada/recent-advances-in-nutritional-immunomodulation-in-dogs-and-cats-53655.

[38] Lam A.T.H., Affolter V.K., Outerbridge C.A., Gericota B., White S.D. (2011). Oral vitamin A as an adjunct treatment for canine sebaceous adenitis. Vet. Dermatol..

[39] Huang Z., Liu Y., Qi G., Brand D., Zheng S.G. (2018). Role of Vitamin A in the Immune System. J. Clin. Med..

[40] Oliveira L.M., Teixeira F.M.E., Sato M.N. (2018). Impact of Retinoic Acid on Immune Cells and Inflammatory Diseases. Mediat. Inflamm..

[41] Roche F.C., Harris-Tryon T.A. (2021). Illuminating the Role of Vitamin A in Skin Innate Immunity and the Skin Microbiome: A Narrative Review. Nutrients.

[42] Van Bennekum A.M., Fisher E.A., Blaner W.S., Harrison E.H. (2000). Hydrolysis of retinyl esters by pancreatic triglyceride lipase. Biochemistry.

[43] Hynd P.I., Hynd P.I. (2019). Digestion in the mono-gastric animal. Animal Nutrition: From Theory to Practice.

[44] Kaser M.M., Hussey C.V., Ellis S.J. (1958). The Absorption of Vitamin A in Dogs Following Cholecystonephrostomy. J. Nutr..

[45] Kouti V., Papazoglou L., Rallis T. (2006). Short-Bowel Syndrome in Dogs and Cats. Compend. Contin. Educ. Pract. Vet..

[46] Wilson D.E., Hejazi J., Elstad N.L., Ing-Fong C., Gleeson J.M., Iverius P.-H. (1987). Novel aspects of vitamin A metabolism in the dog: Distribution of lipoprotein retinyl esters in vitamin A-deprived and cholesterol-fed animals. Biochim. Biophys. Acta.

[47] Chelstowska S., Widjaja-Adhi M.A., Silvaroli J.A., Golczak M. (2016). Molecular Basis for Vitamin A Uptake and Storage in Vertebrates. Nutrients.

[48] Seawright A.A., English P.B., Gartner R.J.W. (1967). Hypervitaminosis A and deforming cervical spondylosis of the cat. J. Comp. Pathol..

[49] Carmona R., Barrena S., Muñoz-Chápuli R. (2019). Retinoids in Stellate Cells: Development, Repair, and Regeneration. J. Dev. Biol..

[50] Chew B.P., Park J.S., Wong T.S., Weng B.C., Kim H.W., Byrne K.M., Hayek M.G., Reinhart G.A. (1998). Role of dietary β-carotene in modulating cell-mediated and humoral immune responses in dogs. FASEB J..

[51] Chew B.P., Park J.S., Weng B.C., Wong T.S., Hayek M.G., Reinhart G.A. (2000). Dietary β-carotene is taken up by blood plasma and leukocytes in dogs. J. Nutr..

[52] Gershoff S.N., Andrus S.B., Hegsted D.M., Lentini E.A. (1957). Vitamin A deficiency in cats. Lab. Investig..

[53] Lakshmanan M.R., Chansang H., Olson J.A. (1972). Purification and properties of carotene 15, 15′-dioxygenase of rabbit intestine. J. Lipid Res..

[54] Schweigert F.J., Raila J., Wichert B., Kienzle E. (2002). Cats absorb beta-carotene, but it is not converted to vitamin A. J. Nutr..

[55] Chew B.P., Park J.S. (2004). Carotenoid action on the immune response. J. Nutr..

[56] Green A.S., Tang G., Lango J., Klasing K.C., Fascetti A.J. (2012). Domestic cats convert [2H8]-β-carotene to [2H4]-retinol following a single oral dose. J. Anim. Physiol. Anim. Nutr..

[57] White H.B., Merrill A.H. (1988). Riboflavin-Binding Proteins. Annu. Rev. Nutr..

[58] M’Clelland D.A. (1996). The Refolding of Riboflavin Binding Protein. Ph.D. Thesis.

[59] van Hoek I., Meyer E., Duchateau L., Peremans K., Smets P., Daminet S. (2009). Retinol-binding protein in serum and urine of hyperthyroid cats before and after treatment with radioiodine. J. Vet. Intern. Med..

[60] Raila J., Brunnberg L., Schweigert F.J., Kohn B. (2010). Influence of kidney function on urinary excretion of albumin and retinol-binding protein in dogs with naturally occurring renal disease. Am. J. Vet. Res..

[61] Steinhoff J.S., Lass A., Schupp M. (2022). Retinoid homeostasis and beyond: How retinol binding protein 4 contributes to health and disease. Nutrients.

[62] Shastak Y., Pelletier W. (2023). Vitamin A supply in swine production: A review of current science and practical considerations. Appl. Anim. Sci..

[63] Schweigert F.J., Ryder O.A., Rambeck W.A., Zucker H. (1990). The majority of vitamin A is transported as retinyl esters in the blood of most carnivores. Comp. Biochem. Physiol. A Comp. Physiol..

[64] Raila J., Buchholz I., Aupperle H., Raila G., Schoon H.A., Schweigert F.J. (2000). The distribution of vitamin A and retinol-binding protein in the blood plasma, urine, liver and kidneys of carnivores. Vet. Res..

[65] Barko P.C., Williams D.A. (2018). Serum concentrations of lipid-soluble vitamins in dogs with exocrine pancreatic insufficiency treated with pancreatic enzymes. J. Vet. Intern. Med..

[66] Raila J., Mathews U., Schweigert F.J. (2001). Plasma transport and tissue distribution of beta-carotene, vitamin A and retinol-binding protein in domestic cats. Comp. Biochem. Physiol. A Mol. Integr. Physiol..

[67] Kolb E., Seehawer J. (2001). Verwertung, Stoffwechsel, Bedeutung und Anwendung des Vitamins A bei Hund und Katze. Prakt. Tierarzt.

[68] Kolb E. (1998). Vitamin A. Verwertung und Anwendung von Vitaminen bei Haustieren.

[69] Schweigert F.J. (1988). Insensitivity of dogs to the effects of nonspecific bound vitamin A in plasma. Int. J. Vitam. Nutr. Res..

[70] Raila J., Radon R., Trüpschuch A., Schweigert F.J. (2002). Retinol and retinyl ester responses in the blood plasma and urine of dogs after a single oral dose of vitamin A. J. Nutr..

[71] Stowe H.D., Lawler D.F., Kealy R.D. (2006). Antioxidant status of pair-fed labrador retrievers is affected by diet restriction and aging. J. Nutr..

[72] Puls R. (1994). Vitamin Levels in Animal Health. Diagnostic Data.

[73] Olsen K., Suri D.J., Davis C., Sheftel J., Nishimoto K., Yamaoka Y., Toya Y., Welham N.V., Tanumihardjo S.A. (2018). Serum retinyl esters are positively correlated with analyzed total liver vitamin A reserves collected from US adults at time of death. Am. J. Clin. Nutr..

[74] O’Byrne S.M., Blaner W.S. (2013). Retinol and retinyl esters: Biochemistry and physiology. J. Lipid Res..

[75] Penniston K.L., Weng N., Binkley N., Tanumihardjo S.A. (2006). Serum retinyl esters are not elevated in postmenopausal women with and without osteoporosis whose preformed vitamin A intakes are high. Am. J. Clin. Nutr..

[76] Chytil F., Ong D.E., Sporn M.B., Roberts A.B., Goodman D.S. (1984). Cellular retinoid-binding proteins In The Retinoids.

[77] Sasaki N., Ishibashi M., Soeta S. (2013). Molecular characterization and tissue distribution of feline retinol-binding protein 4. J. Vet. Med. Sci..

[78] Napoli J.L. (2016). Functions of Intracellular Retinoid Binding-Proteins. Subcell. Biochem..

[79] Levene R., Horton G., Einaugler R. (1964). Retinal Vessel Dehydrogenase Enzymes. Arch. Ophthalmol..

[80] Hoffmann I., Ang H.L., Duester G. (1998). Alcohol dehydrogenases in Xenopus development: Conserved expression of ADH1 and ADH4 in epithelial retinoid target tissues. Dev. Dyn..

[81] Connally H.E., Hamar D.W., Thrall M.A. (2000). Inhibition of canine and feline alcohol dehydrogenase activity by fomepizole. Am. J. Vet. Res..

[82] Kumar S., Sandell L.L., Trainor P.A., Koentgen F., Duester G. (2012). Alcohol and aldehyde dehydrogenases: Retinoid metabolic effects in mouse knockout models. Biochim. Biophys. Acta.

[83] Bchini R., Vasiliou V., Branlant G., Talfournier F., Rahuel-Clermont S. (2013). Retinoic acid biosynthesis catalyzed by retinal dehydrogenases relies on a rate-limiting conformational transition associated with substrate recognition. Chem. Biol. Interact..

[84] Kedishvili N.Y. (2013). Enzymology of retinoic acid biosynthesis and degradation. J. Lipid Res..

[85] Fujihara M., Yamamizu K., Comizzoli P., Wildt D.E., Songsasen N. (2018). Retinoic acid promotes in vitro follicle activation in the cat ovary by regulating expression of matrix metalloproteinase 9. PLoS ONE..

[86] Yang K., Adin C., Shen Q., Lee L.J., Yu L., Fadda P., Samogyi A., Ham K., Xu L., Gilor C. (2017). Aldehyde dehydrogenase 1 a1 regulates energy metabolism in adipocytes from different species. Xenotransplantation.

[87] Harper A.R., Le A.T., Mather T., Burgett A., Berry W., Summers J.A. (2018). Design, synthesis, and ex vivo evaluation of a selective inhibitor for retinaldehyde dehydrogenase enzymes. Bioorganic Med. Chem..

[88] Cho K., Lee S.-M., Heo J., Kwon Y.M., Chung D., Yu W.-J., Bae S.S., Choi G., Lee D.-S., Kim Y. (2021). Retinaldehyde Dehydrogenase Inhibition-Related Adverse Outcome Pathway: Potential Risk of Retinoic Acid Synthesis Inhibition during Embryogenesis. Toxins.

[89] Kasimanickam V.R. (2016). Expression of retinoic acid-metabolizing enzymes, ALDH1A1, ALDH1A2, ALDH1A3, CYP26A1, CYP26B1 and CYP26C1 in canine testis during post-natal development. Reprod. Domest. Anim..

[90] Balmer J.E., Blomhoff R. (2002). Gene expression regulation by retinoic acid. J. Lipid Res..

[91] Brandebourg T.D., Hu C.Y. (2005). Regulation of differentiating pig preadipocytes by retinoic acid. J. Anim. Sci..

[92] de Mello Souza C.H., Valli V.E., Kitchell B.E. (2014). Detection of retinoid receptors in non-neoplastic canine lymph nodes and in lymphoma. Can. Vet. J..

[93] Hoffmann B., Lehmann J.M., Zhang X.K., Hermann T., Husmann M., Graupner G., Pfahl M. (1990). A retinoic acid receptor-specific element controls the retinoic acid receptor-beta promoter. Mol. Endocrinol..

[94] Rastinejad F., Wagner T., Zhao Q., Khorasanizadeh S. (2000). Structure of the RXR-RAR DNA-binding complex on the retinoic acid response element DR1. EMBO J..

[95] Huang P., Chandra V., Rastinejad F. (2014). Retinoic acid actions through mammalian nuclear receptors. Chem. Rev..

[96] Wolf G. (2004). The visual cycle of the cone photoreceptors of the retina. Nutr. Rev..

[97] Wang J.S., Kefalov V.J. (2011). The cone-specific visual cycle. Prog. Retin. Eye Res..

[98] Byosiere S.E., Chouinard P.A., Howell T.J., Bennett P.C. (2018). What do dogs (*Canis familiaris*) see? A review of vision in dogs and implications for cognition research. Psychon. Bull. Rev..

[99] Kemp C.M., Jacobson S.G., Borruat F.X., Chaitin M.H. (1989). Rhodopsin levels and retinal function in cats during recovery from vitamin A deficiency. Exp. Eye Res..

[100] Miyazono S., Isayama T., Delori F.C., Makino C.L. (2011). Vitamin A activates rhodopsin and sensitizes it to ultraviolet light. Vis. Neurosci..

[101] Palczewski K. (2014). Chemistry and biology of the initial steps in vision: The Friedenwald lecture. Investig. Ophthalmol. Vis. Sci..

[102] Clagett-Dame M., Knutson D. (2011). Vitamin A in Reproduction and Development. Nutrients.

[103] Kono M., Goletz P.W., Crouch R.K. (2008). 11-cis- and all-trans-retinols can activate rod opsin: Rational design of the visual cycle. Biochemistry.

[104] Kiser P.D. (2022). Retinal pigment epithelium 65 kDa protein (RPE65): An update. Prog. Retin. Eye Res..

[105] Stecher H., Gelb M.H., Saari J.C., Palczewski K. (1999). Preferential release of 11-cis-retinol from retinal pigment epithelial cells in the presence of cellular retinaldehyde-binding protein. J. Biol. Chem..

[106] Parker R.O., Crouch R.K. (2010). Retinol dehydrogenases (RDHs) in the visual cycle. Exp. Eye Res..

[107] Strauss O. (2005). The retinal pigment epithelium in visual function. Physiol. Rev..

[108] Daruwalla A., Choi E.H., Palczewski K., Kiser P.D. (2018). Structural biology of 11-cis-retinaldehyde production in the classical visual cycle. Biochem. J..

[109] Palczewski K., Kiser P.D. (2020). Shedding new light on the generation of the visual chromophore. Proc. Natl. Acad. Sci. USA.

[110] Shastak Y., Pelletier W. (2023). Nutritional Balance Matters: Assessing the Ramifications of Vitamin A Deficiency on Poultry Health and Productivity. Poultry.

[111] Dawson H.D., Collins G., Pyle R., Key M., Taub D.D. (2008). The Retinoic Acid Receptor-alpha mediates human T-cell activation and Th2 cytokine and chemokine production. BMC Immunol..

[112] Mora J.R., Iwata M., von Andrian U.H. (2008). Vitamin effects on the immune system: Vitamins A and D take centre stage. Nat. Rev. Immunol..

[113] Matikainen S., Ronni T., Hurme M., Pine R., Julkunen I. (1996). Retinoic acid activates interferon regulatory factor-1 gene expression in myeloid cells. Blood.

[114] Al Tanoury Z., Piskunov A., Rochette-Egly C. (2013). Vitamin A and retinoid signaling: Genomic and nongenomic effects. J. Lipid Res..

[115] Hao X., Chen H., Li Y., Chen B., Liang W., Xiao X., Zhou P., Li S. (2022). Molecular characterization and antiviral effects of canine interferon regulatory factor 1 (CaIRF1). BMC Vet. Res..

[116] Liu Y., Liu X., Kang H., Hu X., Liu J., Tian J., Qu L. (2018). Identification of Feline Interferon Regulatory Factor 1 as an Efficient Antiviral Factor against the Replication of Feline Calicivirus and Other Feline Viruses. Biomed. Res. Int..

[117] Jefferies C.A. (2019). Regulating IRFs in IFN Driven Disease. Front. Immunol..

[118] Tourkochristou E., Triantos C., Mouzaki A. (2021). The Influence of Nutritional Factors on Immunological Outcomes. Front. Immunol..

[119] Green H.N., Mellanby E. (1928). Vitamin A as an anti-infective agent. Brit. Med. J..

[120] Bono M.R., Tejon G., Flores-Santibañez F., Fernandez D., Rosemblatt M., Sauma D. (2016). Retinoic Acid as a Modulator of T Cell Immunity. Nutrients.

[121] Villamor E., Fawzi W. (2005). Effects of Vitamin A supplementation on immune responses and correlation with clinical outcomes. Clin. Microbiol. Rev..

[122] Gürbüz M., Aktaç S. (2022). Understanding the role of Vitamin A and its precursors in the immune system. Nutr. Clin. Metab..

[123] Stephensen C.B. (2001). Vitamin A, infection, and immune function. Annu. Rev. Nutr..

[124] Hu Y., Zhang L., Zhang Y., Xiong H., Wang F., Wang Y., Lu Z. (2020). Effects of starch and gelatin encapsulated Vitamin A on growth performance, immune status and antioxidant capacity in weaned piglets. Anim. Nutr..

[125] Ross S.A., McCaffery P.Y., Drager U.C., de Luca L.M. (2000). Retinoids in embryonal development. Physiol. Rev..

[126] Kam R.K., Deng Y., Chen Y., Zhao H. (2012). Retinoic acid synthesis and functions in early embryonic development. Cell Biosci..

[127] Duong V., Rochette-Egly C. (2011). The molecular physiology of nuclear retinoic acid receptors. From health to disease. Biochim. Biophys. Acta.

[128] Dubey A., Rose R.E., Jones D.R., Saint-Jeannet J.P. (2018). Generating retinoic acid gradients by local degradation during craniofacial development: One cell’s cue is another cell’s poison. Genesis.

[129] Brown G. (2023). Retinoic acid receptor regulation of decision-making for cell differentiation. Front. Cell Dev. Biol..

[130] Duester G. (2008). Retinoic acid synthesis and signaling during early organogenesis. Cell.

[131] Qiu J., Nordling S., Vasavada H.H., Butcher E.C., Hirschi K.K. (2020). Retinoic Acid Promotes Endothelial Cell Cycle Early G1 State to Enable Human Hemogenic Endothelial Cell Specification. Cell Rep..

[132] Organisation for Economic Co-operation and Development (OECD) Detailed Review Paper on the Retinoid System. OECD Series on Testing and Assessment, Nr.343, Paris. 2021. https://one.oecd.org/document/ENV/CBC/MONO(2021)20/en/pdf.

[133] Gross K.L., Wedekind K.J., Kirk C.A., Cowell C.S., Schoenherr W.D., Richardson D.C. (1990). Effect of dietary vitamin A on reproductive performance and immune response of dogs. Am. J. Vet. Res..

[134] Werner A.H., Power H.T. (1994). Retinoids in veterinary dermatology. Clin. Dermatol..

[135] Pauli S.A., Session D.R., Shang W., Easley K., Wieser F., Taylor R.N., Pierzchalski K., Napoli J.L., Kane M.A., Sidell N. (2013). Analysis of follicular fluid retinoids in women undergoing in vitro fertilization: Retinoic acid influences embryo quality and is reduced in women with endometriosis. Reprod. Sci..

[136] Demczuk M., Huang H., White C., Kipp J.L. (2016). Retinoic acid regulates calcium signaling to promote mouse ovarian granulosa cell proliferation. Biol. Reprod..

[137] Suwa H., Kishi H., Imai F., Nakao K., Hirakawa T., Minegishi T. (2016). Retinoic acid enhances progesterone production via the cAMP/PKA signaling pathway in immature rat granulosa cells. Biochem. Biophys. Rep..

[138] Jiang Y., Li C., Chen L., Wang F., Zhou X. (2017). Potential role of retinoids in ovarian physiology and pathogenesis of polycystic ovary syndrome. Clin. Chim. Acta.

[139] Munetsuna E., Hojo Y., Hattori M., Ishii H., Kawato S., Ishida A., Kominami S.A., Yamazaki T. (2009). Retinoic acid stimulates 17β-estradiol and testosterone synthesis in rat hippocampal slice cultures. Endocrinology.

[140] Damdimopoulou P., Chiang C., Flaws J.A. (2019). Retinoic acid signaling in ovarian folliculogenesis and steroidogenesis. Reprod. Toxicol..

[141] Han B.C., Xia H.F., Sun J., Yang Y., Peng J.P. (2010). Retinoic acid-metabolizing enzyme cytochrome P450 26a1 (cyp26a1) is essential for implantation: Functional study of its role in early pregnancy. J. Cell. Physiol..

[142] Yin Y., Haller M.E., Chadchan S.B., Kommagani R., Ma L. (2021). Signaling through retinoic acid receptors is essential for mammalian uterine receptivity and decidualization. JCI Insight.

[143] Crespo D., Assis L.H.C., van de Kant H.J.G., de Waard S., Safian D., Lemos M.S., Bogerd J., Schulz R.W. (2019). Endocrine and local signaling interact to regulate spermatogenesis in zebrafish: Follicle-stimulating hormone, retinoic acid and androgens. Development.

[144] Griswold M.D. (2022). Cellular and molecular basis for the action of retinoic acid in spermatogenesis. J. Mol. Endocrinol..

[145] Jewell D.E., Toll P.W., Wedekind K.J., Zicker S.C. (2000). Effect of increasing dietary antioxidants on concentrations of vitamin E and total alkenals in serum of dogs and cats. Vet. Ther..

[146] Espadas I., Ricci E., McConnell F., Sanchez-Masian D. (2017). MRI, CT and histopathological findings in a cat with hypovitaminosis A. Vet. Rec. Case Rep..

[147] Landete J.M. (2013). Dietary intake of natural antioxidants: Vitamins and polyphenols. Crit. Rev. Food Sci. Nutr..

[148] Krinsky N.I., Johnson E.J. (2005). Carotenoid actions and their relation to health and disease. Mol. Asp. Med..

[149] Dao D.Q., Ngo T.C., Thong N.M., Nam P.C. (2017). Is vitamin A an antioxidant or a pro-oxidant?. J. Phys. Chem. B.

[150] Palace V.P., Khaper N., Qin Q., Singal P.K. (1999). Antioxidant potentials of vitamin A and carotenoids and their relevance to heart disease. Free Radic. Biol. Med..

[151] Blaner W.S., Shmarakov I.O., Traber M.G. (2021). Vitamin A and vitamin E: Will the real antioxidant please stand up?. Annu. Rev. Nutr..

[152] Park U.-H., Han H.S., Um E., An X.-H., Kim E.-J., Um S.J. (2009). Redox regulation of transcriptional activity of retinoic acid receptor by thioredoxin glutathione reductase (TGR). Biochem. Biophys. Res. Commun..

[153] El Haddad M., Jean E., Turki A., Hugon G., Vernus B., Bonnieu A., Passerieux E., Hamade A., Mercier J., Laoudj-Chenivesse D. (2012). Glutathione peroxidase 3, a new retinoid target gene, is crucial for human skeletal muscle precursor cell survival. J. Cell Sci..

[154] Brigelius-Flohé R., Flohé L. (2020). Regulatory phenomena in the glutathione peroxidase superfamily. Antioxid. Redox Signal..

[155] Rao J., Zhang C., Wang P., Lu L., Zhang F. (2010). All-trans retinoic acid alleviates hepatic ischemia/reperfusion injury by enhancing manganese superoxide dismutase in rats. Biol. Pharm. Bull..

[156] Azzam M.M., Hussein A.M., Marghani B.H., Barakat N.M., Khedr M.M.M., Heakel N.A. (2022). Retinoic acid potentiates the therapeutic efficiency of bone marrow-derived mesenchymal stem cells (BM-MSCs) against cisplatin-induced hepatotoxicity in rats. Sci. Pharm..

[157] Tourniaire F., Musinovic H., Gouranton E., Astier J., Marcotorchino J., Arreguin A., Bernot D., Palou A., Bonet M.L., Ribot J. (2015). All-trans retinoic acid induces oxidative phosphorylation and mitochondria biogenesis in adipocytes. J. Lipid Res..

[158] Tripathy S., Chapman J.D., Han C.Y., Hogarth C.A., Arnold S.L., Onken J., Kent T., Goodlett D.R., Isoherranen N. (2016). All-trans-retinoic acid enhances mitochondrial function in models of human liver. Mol. Pharmacol..

[159] Rajawat Y., Hilioti Z., Bossis I. (2010). Autophagy: A target for retinoic acids. Autophagy.

[160] Choi A.M., Ryter S.W., Levine B. (2013). Autophagy in human health and disease. N. Engl. J. Med..

[161] Polizopoulou Z.S., Kazakos G., Patsikas M.N., Roubies N. (2005). Hypervitaminosis A in the cat: A case report and review of the literature. J. Feline Med. Surg..

[162] Guerra J.M., Daniel A.G., Aloia T.P., de Siqueira A., Fukushima A.R., Simões D.M.N., Reche-Júior A., Cogliati B. (2014). Hypervitaminosis A-induced hepatic fibrosis in a cat. Educ. Eval. Policy Anal..

[163] Ralli E.P., Pariente A., Flaum G., Waterhouse A. (1933). A study of vitamin A deficiency in normal and depancreatized dogs. Am. J. Physiol.-Leg. Content.

[164] Crimm P.D., Short D.M. (1937). Vitamin a deficiency in the dog. Am. J. Physiol.-Leg. Content.

[165] Wennogle S.A., Priestnall S.L., Suárez-Bonnet A., Webb C.B. (2019). Comparison of clinical, clinicopathologic, and histologic variables in dogs with chronic inflammatory enteropathy and low or normal serum 25-hydroxycholecalciferol concentrations. J. Vet. Intern. Med..

[166] Olcott H.S. (1933). Vitamin A Deficiency in the Dog. Proc. Soc. Exp. Biol. Med..

[167] Ihrke P.J., Goldschmidt M.H. (1983). Vitamin A-responsive dermatosis in the dog. J. Am. Vet. Med. Assoc..

[168] Verstegen J., Dhaliwal G., Verstegen-Onclin K. (2008). Canine and feline pregnancy loss due to viral and non-infectious causes: A review. Theriogenology.

[169] Glover J. (1983). Factors affecting vitamin A transport in animals and man. Proc. Nutr. Soc..

[170] Olson J.A. (1984). Serum levels of vitamin A and carotenoids as reflectors of nutritional status. J. Natl. Cancer Inst..

[171] Schweigert F.J., Thomann E., Zucker H. (1991). Vitamin A in the urine of carnivores. Int. J. Vitam. Nutr. Res..

[172] Faure H., Preziosi P., Roussel A.M., Bertrais S., Galan P., Hercberg S., Favier A. (2006). Factors influencing blood concentration of retinol, α-tocopherol, vitamin C, and β-carotene in the French participants of the SU.VI.MAX trial. Eur. J. Clin. Nutr..

[173] Ross A., Harrison E., Zempleni J., Rucker R.B., Suttie J.W., McCormick D.B. (2007). Vitamin A: Nutritional aspects of retinoids and carotenoids. Handbook of Vitamins.

[174] Shastak Y., Witzig M., Hartung K., Bessei W., Rodehutscord M. (2012). Comparison and evaluation of bone measurements for the assessment of mineral phosphorus sources in broilers. Poult. Sci..

[175] Shastak Y., Rodehutscord M. (2013). Determination and estimation of phosphorus availability in growing poultry and their historical development. World’s Poult. Sci. J..

[176] Huang J., Weinstein S.J., Yu K., Männistö S., Albanes D. (2021). Association between serum retinol and overall and cause-specific mortality in a 30-year prospective cohort study. Nat. Commun..

[177] Fascetti A.J. (2010). Nutritional management and disease prevention in healthy dogs and cats. R. Bras. Zootec..

[178] Cline M.G., Burns K.M., Coe J.B., Downing R., Durzi T., Murphy M., Parker V. (2021). 2021 AAHA Nutrition and Weight Management Guidelines for Dogs and Cats. J. Am. Anim. Hosp. Assoc..

[179] Clark L. (1971). Hypervitaminosis A: A review. Aust. Vet. J..

[180] Kolb E. (1999). Vitamin A. Der Gehalt an Vitaminen im Blut, im Blutplasma, in Geweben und in der Milch von Haustieren: Bedeutung für Gesundheit und Diagnostik.

[181] EFSA (European Food Safety Authority) (2015). Scientific Opinion on Dietary Reference Values for vitamin A. EFSA J..

[182] Seawright A.A., Hrdlicka J. (1974). Pathogenetic factors in tooth loss in young cats on a high daily oral intake of vitamin A. Aust. Vet. J..

[183] NASEM (The National Academies of Sciences, Engineering, and Medicine) (1987). Vitamin Tolerance of Animals.

[184] AAFCO (Association of American Feed Control Officials). 2014. AAFCO Dog and Cat Food Nutrient Profiles, Champaign, Illinois, USA. https://www.aafco.org/wp-content/uploads/2023/01/Model_Bills_and_Regulations_Agenda_Midyear_2015_Final_Attachment_A.__Proposed_revisions_to_AAFCO_Nutrient_Profiles_PFC_Final_070214.pdf.

[185] Zafalon R.V.A., Ruberti B., Rentas M.F., Amaral A.R., Vendramini T.H.A., Chacar F.C., Kogika M.M., Brunetto M.A. (2020). The Role of Vitamin D in Small Animal Bone Metabolism. Metabolites.

[186] Džopalić T., Božić-Nedeljković B., Jurišić V. (2021). The role of vitamin A and vitamin D in modulation of the immune response with a focus on innate lymphoid cells. Cent. Eur. J. Immunol..

[187] Guilland J.-C. (2011). Les interactions entre les vitamines A, D, E et K: Synergie et/ou competition. OCL.

[188] MacDonald P.N., Dowd D.R., Nakajima S., Galligan M.A., Reeder M.C., Haussler C.A., Ozato K., Haussler M.R. (1993). Retinoid X receptors stimulate and 9-cis retinoic acid inhibits 1,25-dihydroxyvitamin D3-activated expression of the rat osteocalcin gene. Mol. Cell. Biol..

[189] Ruiter B., Patil S.U., Shreffler W.G. (2015). Vitamins A and D have antagonistic effects on expression of effector cytokines and gut-homing integrin in human innate lymphoid cells. Clin. Exp. Allergy.

[190] Cheng Y.H., Chiang E.P., Syu J.N., Chao C.Y., Lin H.Y., Lin C.C., Yang M.D., Tsai S.Y., Tang F.Y. (2021). Treatment of 13-cis retinoic acid and 1,25-dihydroxyvitamin D3 inhibits TNF-alpha-mediated expression of MMP-9 protein and cell invasion through the suppression of JNK pathway and microRNA 221 in human pancreatic adenocarcinoma cancer cells. PLoS ONE.

[191] Treptow S., Grün J., Scholz J., Radbruch A., Heine G., Worm M. (2021). 9-cis Retinoic acid and 1.25-dihydroxyvitamin D3 drive differentiation into IgA+ secreting plasmablasts in human naïve B cells. Eur. J. Immunol..

[192] Shastak Y., Obermueller-Jevic U., Pelletier W. (2023). A Century of Vitamin E: Early Milestones and Future Directions in Animal Nutrition. Agriculture.

[193] Tesoriere L., Bongiorno A., Pintaudi A.M., D’Anna R., D’Arpa D., Livrea M.A. (1996). Synergistic interactions between vitamin A and vitamin E against lipid peroxidation in phosphatidylcholine liposomes. Arch. Biochem. Biophys..

[194] Tesoriere L., Ciaccio M., Bongiorno A., Riccio A., Pintaudi A.M., Livrea M.A. (1993). Antioxidant activity of all-trans-retinol in homogeneous solution and in phosphatidylcholine liposomes. Arch. Biochem. Biophys..

[195] De Tullio M.C. (2010). The Mystery of Vitamin C. Nat. Educ..

[196] Kanter M., Coskun O., Armutcu F., Uz Y.H., Kizilay G. (2005). Protective effects of vitamin C, alone or in combination with vitamin A, on endotoxin-induced oxidative renal tissue damage in rats. Tohoku J. Exp. Med..

[197] Hosseini Omshi F.S., Abbasalipourkabir R., Abbasalipourkabir M., Nabyan S., Bashiri A., Ghafourikhosroshahi A. (2018). Effect of vitamin A and vitamin C on attenuation of ivermectin-induced toxicity in male Wistar rats. Environ. Sci. Pollut. Res. Int..

[198] Lin P.H., Sermersheim M., Li H., Lee P.H.U., Steinberg S.M., Ma J. (2017). Zinc in Wound Healing Modulation. Nutrients.

[199] Rahman M.M., Wahed M.A., Fuchs G.J., Baqui A.H., Alvarez J.O. (2002). Synergistic Effect of Zinc and Vitamin A on the Biochemical Indexes of Vitamin A Nutrition in Children. Am. J. Clin. Nutr..

[200] Hernandez F.M.O., Santos M.O., Venturin G.L., Bragato J.P., Rebech G.T., Melo L.M., Costa S.F., de Freitas J.H., Siqueira C.E., Morais D.A. (2021). Vitamins A and D and Zinc Affect the Leshmanicidal Activity of Canine Spleen Leukocytes. Animals.

[201] Jackson C., Kolba N., Tako E. (2023). Assessing the Interactions between Zinc and Vitamin A on Intestinal Functionality, Morphology, and the Microbiome In Vivo (*Gallus gallus*). Nutrients.

[202] Long K.Z., Rosado J.L., Montoya Y., Solano M.D.L., Hertzmark E., DuPont H.L., Santos J.I. (2007). Effect of Vitamin A and Zinc Supplementation on Gastrointestinal Parasitic Infections among Mexican Children. Pediatrics.

[203] Smith J.C. (1980). The vitamin A-zinc connection: A review. Ann. N. Y. Acad. Sci..

[204] Christian P., West K.P. (1998). Interactions between zinc and vitamin A: An update. Am. J. Clin. Nutr..

[205] Ahn J., Koo S.I. (1995). Effects of zinc and essetial fatty acid deficiencies on the lymphatic absorption of vitamin A and secretion of phospholipids. J. Nutr. Biochem..

[206] Shastak Y., Pelletier W. (2023). Balancing Vitamin A Supply for Cattle: A Review of the Current Knowledge. Advances in Animal Science and Zoology 21.

[207] Shastak Y., Pelletier W. (2023). Delving into Vitamin A Supplementation in Poultry Nutrition: Current Knowledge, Functional Effects, and Practical Implications. Worlds Poult. Sci. J..

[208] Shearin A.L., Ostrander E.A. (2010). Canine morphology: Hunting for genes and tracking mutations. PLoS Biol..

[209] Streitberger K., Schweizer M., Kropatsch R., Dekomien G., Distl O., Fischer M.S., Epplen J.T., Hertwig S.T. (2012). Rapid genetic diversification within dog breeds as evidenced by a case study on Schnauzers. Anim. Genet..

[210] Finka L.R., Luna S.P.L., Mills D.S., Farnworth M.J. (2020). The Application of Geometric Morphometrics to Explore Potential Impacts of Anthropocentric Selection on Animals’ Ability to Communicate via the Face: The Domestic Cat as a Case Study. Front. Vet. Sci..

[211] Friedrich J., Talenti A., Arvelius P., Strandberg E., Haskell M.J., Wiener P. (2020). Unravelling selection signatures in a single dog breed suggests recent selection for morphological and behavioral traits. Adv. Genet..

[212] Lauridsen M. (2016). Vitamin E—A Key Nutritional Factor for Health and Growth.

[213] Sacakli P., Ramay M.S., Harijaona J.A., Shastak Y., Pelletier W., Gordillo A., Calik A. 2024. Does the latest NASEM vitamin A requirement estimate support optimal growth in broiler chickens?. Proceedings of the 16th European Poultry Congress.

[214] Kim J., Williams F.J., Dreger D.L., Plassais J., Davis B.W., Parker H.G., Ostrander E.A. (2018). Genetic selection of athletic success in sport-hunting dogs. Proc. Natl. Acad. Sci. USA.

[215] Meyer H., Heckötter E. (1986). Futterwerttabellen für Hunde und Katzen.

[216] McDowell L.R. (2000). Vitamin A. Vitamins in Animal and Human Nutrition.

[217] McDowell L.R. (1989). Vitamin Supplementation. Vitamins in Animal Nutrition: Comparative Aspects to Human Nutrition.

[218] Kırkpınar F., Açıkgöz Z., Yücel B., Taşkin T. (2018). Feeding. Animal Husbandry and Nutrition.

[219] AWT (Arbeitsgemeinschaft für Wirkstoffe in der Tierernährung e.V.) (2002). Vitamins in Animal Nutrition: Vitamin E.

[220] Xu Y., Shrestha N., Préat V., Beloqui A. (2021). An overview of in vitro, ex vivo and in vivo models for studying the transport of drugs across intestinal barriers. Adv. Drug Deliv. Rev..

[221] King A.J. (2012). The use of animal models in diabetes research. Br. J. Pharmacol..

[222] Moro C.A., Hanna-Rose W. (2020). Animal Model Contributions to Congenital Metabolic Disease. Adv. Exp. Med. Biol..

[223] Campion S., Inselman A., Hayes B., Casiraghi C., Joseph D., Facchinetti F., Salomone F., Schmitt G., Hui J., Davis-Bruno K. (2022). The benefits, limitations and opportunities of preclinical models for neonatal drug development. Dis. Model. Mech..

[224] Borel P., Desmarchelier C. (2017). Genetic Variations Associated with Vitamin A Status and Vitamin A Bioavailability. Nutrients.

[225] Suzuki M., Tomita M. (2022). Genetic Variations of Vitamin A-Absorption and Storage-Related Genes, and Their Potential Contribution to Vitamin A Deficiency Risks Among Different Ethnic Groups. Front. Nutr..

[226] Tanumihardjo S.A. (2011). Vitamin A: Biomarkers of nutrition for development. Am. J. Clin. Nutr..

[227] Tanumihardjo S.A., Russell R.M., Stephensen C.B., Gannon B.M., Craft N.E., Haskell M.J., Lietz G., Schulze K., Raiten D.J. (2016). Biomarkers of Nutrition for Development (BOND)-Vitamin A Review. J. Nutr..

[228] Amimo J.O., Michael H., Chepngeno J., Raev S.A., Saif L.J., Vlasova A.N. (2022). Immune Impairment Associated with Vitamin A Deficiency: Insights from Clinical Studies and Animal Model Research. Nutrients.

[229] Carratù M.R., Marasco C., Mangialardi G., Vacca A. (2012). Retinoids: Novel immunomodulators and tumour-suppressive agents?. Br. J. Pharmacol..

[230] Bos A., van Egmond M., Mebius R. (2022). The role of retinoic acid in the production of immunoglobulin A. Mucosal Immunol..

[231] Stokes C., Waly N. (2006). Mucosal defence along the gastrointestinal tract of cats and dogs. Vet. Res..

[232] Allenspach K. (2011). Clinical immunology and immunopathology of the canine and feline intestine. Vet. Clin. N. Am. Small Anim. Pract..

[233] Petersen-Jones S.M., Komáromy A.M. (2024). Canine and Feline Models of Inherited Retinal Diseases. Cold Spring Harb. Perspect. Med..

[234] Mooney A. (2016). Stability of Essential Nutrients in Pet Food Manufacturing and Storage. Master’s Thesis.

[235] Belay T., Lambrakis L., Goodgame S., Price A., Kersey J., Shields R. (2018). 392 Vitamin Stability in wet pet food Formulation and Production perspective. J. Anim. Sci..

[236] Shastak Y., Pelletier W. (2024). A cross-species perspective: β-carotene supplementation effects in poultry, swine, and cattle—A review. J.Appl. Anim. Res..

[237] Galli G.M., Andretta I., Martinez N., Wernick B., Shastak Y., Gordillo A., Gobi J. (2024). Stability of vitamin A at critical points in pet-feed manufacturing and during premix storage. Front. Vet. Sci..

